# Production and Optimization of Anti-Aging Peptides from *Pleurotus eryngii* Mushroom Feet: Mechanistic Insights via Integrated Transcriptomics and Metabolomics

**DOI:** 10.3390/foods14223977

**Published:** 2025-11-20

**Authors:** Shangmeng Wang, Haiyan Li, Fen Zhao, Ji’an Gao, Shuaishuai Huang, Xinqi Liu, Biao Ma

**Affiliations:** 1 Key Laboratory of Geriatric Nutrition and Health, Beijing Technology and Business University (BTBU), Beijing 100048, China; 2 China Food Flavor and Nutrition Health Innovation Center, Beijing Technology and Business University (BTBU), Beijing 100048, China; 3 School of Food and Healthy, Beijing Technology and Business University (BTBU), Beijing 100048, China; 4 Beijing Science Sun Pharmaceutical Co., Ltd., Beijing 100176, China

**Keywords:** *Pleurotus eryngii* mushroom feet, peptides, response surface methodology, senescence, transcriptomics, LC–MS-based metabolomics, PC12 cell model

## Abstract

This study established an optimized process for obtaining anti-aging peptides from *Pleurotus eryngii* mushroom feet (PEMFPeps). Using response surface methodology, high yields of protein (51.31 ± 3.00%) and peptides (48.71 ± 0.17% hydrolysis degree) were achieved. In a D-galactose-induced PC12 cell aging model, the simulated digests (SID-PEMFPeps) exhibited potent anti-aging effects at a concentration of 1 mg/mL. An integrated transcriptomic and metabolomic approach was employed to systematically investigate the underlying mechanisms. The results revealed that Integrated transcriptomic and metabolomic analyses showed that SID-PEMFPeps alleviated cellular senescence through multi-dimensional regulation of transcriptional and metabolic networks. This included modulating key pathways related to oxidative stress, synaptic function, and energy metabolism (e.g., glutamatergic synapse, pentose phosphate pathway, and TCA cycle), and reversing the aberrant expression of aging-associated genes (e.g., *Arap3, Grin2d and Npy1r*). Our findings demonstrate that SID-PEMFPeps are promising candidates for functional foods targeting age-related dysfunction though their efficacy and safety in vivo require further validation.

## 1. Introduction

*Pleurotus eryngii*, commonly known as the king oyster mushroom, is one of the most widely cultivated edible mushrooms globally, prized for its unique texture, flavor, and nutritional profile [1]. It is rich in protein, polysaccharides, and dietary fiber [2, 3, 4, 5]. For instance, Nie et al. reported that *Pleurotus eryngii* powder contains 16.39% protein, 35.45% polysaccharides, and 34.03% dietary fiber [6]. While polysaccharides have traditionally been the focus of research, recent attention has shifted towards their protein components and the potential to derive bioactive peptides, positioning this as an emerging research hotspot.

The rapid expansion of the edible mushroom industry has led to a substantial generation of by-products, such as mushroom feet, heads, and misshapen fruiting bodies, which can account for up to 20% of the total biomass [7]. These by-products possess a nutritional composition similar to that of commercial fruiting bodies, with a crude protein content of approximately 20.69% [7]. However, they are often discarded, representing a significant waste of resources and a missed opportunity for value-added utilization [8].

The production of bioactive peptides from these underutilized sources offers a promising strategy for sustainable valorization. Bioactive peptides derived from edible mushrooms have been shown to exhibit significant health-promoting effects. For example, peptides from *Auricularia auricula* were shown to enhance antioxidant enzyme activities and suppress H_2_O_2_-induced senescence in HepG2 cells [9]. Antioxidant and anti-inflammatory peptides from *Ganoderma lucidum* and *Tricholoma matsutake* have also been identified, with mechanisms linked to the downregulation of the NF-κB and NLRP3 inflammasome pathways [10, 11, 12, 13]. In our previous study, we contributed to this field by demonstrating that simulated in vitro-digested peptides from *Pleurotus eryngii* mushroom feet (SID-PEMFPeps) delayed D-galactose-induced senescence in PC12 cells by modulating the TLR4/NF-κB/MAPK signaling pathway [14].

The efficacy of protein-derived peptides is closely dependent on the extraction and hydrolysis processes employed. Although alkali extraction–acid precipitation methods are efficient, they often lead to protein denaturation [15, 16]. Enzymatic extraction and membrane separation provide gentler alternatives but face challenges related to cost, scalability, and potential structural alteration [17, 18, 19]. In contrast, salt solubilization assisted by ultrasound represents a milder and more efficient approach, better preserving protein structure and functionality [20, 21, 22]. Subsequent enzymatic hydrolysis, influenced by factors like protease type, pH, temperature, and time, is critical for generating bioactive peptide sequences [23]. Therefore, optimizing these upstream processes is paramount to maximizing the yield and bioactivity of the final product.

Building on our previous discovery of the TLR4/NF-κB/MAPK pathway involvement [14], a pivotal question arises: is this the sole mechanism, or does the anti-aging effect of SID-PEMFPeps involve a broader, system-wide reprogramming of cellular functions? Aging is a complex process characterized by multifaceted molecular dysfunction. We therefore hypothesized that SID-PEMFPeps confer their anti-aging benefits through a multi-dimensional mechanism that orchestrates extensive changes in gene expression and metabolic homeostasis, extending beyond a single signaling cascade. To comprehensively test this hypothesis, we employed an integrated transcriptomics and metabolomics strategy, which provides an unbiased, systems-level perspective to map the global molecular networks modulated by the peptides.

To test this hypothesis and bridge the knowledge gap, our study was designed with two interconnected objectives. First, we established a robust and efficient production pipeline by systematically optimizing the extraction of *Pleurotus eryngii* mushroom feet protein (PEMFP) using ultrasound-assisted salt solubilization and its enzymatic hydrolysis into peptides (PEMFPeps) using response surface methodology (RSM). This ensures the preparation of a high-quality, consistent substrate for biological evaluation. Second, we employed an integrated transcriptomics and metabolomics approach to systematically investigate the effects of SID-PEMFPeps on a D-galactose-induced aging model in PC12 cells. This unbiased, systems-level strategy allows us to move beyond targeted hypothesis testing and comprehensively map the global molecular networks and key regulatory nodes modulated by SID-PEMFPeps. A schematic flowchart illustrating the overall experimental design is provided in Supplementary Figure S1.

In this study, an optimized extraction process was established for obtaining anti-aging peptides from *Pleurotus eryngii* mushroom feet (PEMFPeps), achieving high yields of protein (51.31 ± 3.00%) and peptides (48.71 ± 0.17%). Furthermore, integrated transcriptomic and metabolomic analyses revealed that PEMFPeps mitigated cellular senescence by regulating oxidative stress, synaptic signaling, and energy metabolism pathways, including the glutamatergic synapse and TCA cycle. These findings provide a scientific basis for the valorization of mushroom by-products and suggest that PEMFPeps may serve as promising bioactive ingredients for functional foods targeting age-related dysfunction, though further in vivo studies are needed to confirm their bioactivity.

## 2. Materials and Methods

### 2.1. Materials and Chemicals

*Pleurotus eryngii* mushroom feet were obtained from Xuzhou, China. *Pleurotus eryngii* grows in an environment with temperatures ranging from 18–25 °C and humidity between 80%–90%, and mushrooms were harvested at the same developmental stage to minimize variability. Throughout the study, the same batch of materials was used to ensure consistency. Alkaline protease, pepsin, D-Galactose (D-Gal), and TRIzol^®^ reagents were procured from Shanghai YuanYe Biological Technology Co., Ltd. (Shanghai, China). Dialysis bags of molecular weight cutoff (MWCO) 200 Da were acquired from Microdyn-Nadir GmbH (Microdyn-Nadir GmbH, Wiesbaden, Germany). PC12 cells (4th generation) were procured from Shanghai Fuheng Biological Technology Co., Ltd. (Shanghai, China). RPMI 1640 medium (1640), penicillin-streptomycin (PS), phosphate-buffered saline (PBS), and trypsin-EDTA (TE) were procured from Gibco (Grand Island, NE, USA). Fetal bovine serum (FBS) was obtained from Excell Biotechnology Co. (Taicang, China). Horse serum (HS), RIPA lysis buffer, BCA protein concentration determination kit were procured from Beijing Solarbio Science & Technology Co., Ltd. (Beijing, China), and Cell Counting Kit-8 (CCK-8) kit were sourced from Beyotime Biotechnology Co., Ltd. (Shanghai, China). The LC-MS/MS analysis of the metabolic profiles was performed using a Thermo Fisher Scientific UHPLC-Q Exactive HF-X System (Thermo Fisher Scientific, Waltham, MA, USA) to obtain detailed mass spectrometry data for the sample.

### 2.2. Nutritional Composition Determination of Pleurotus eryngii and PEMFPeps

The determination of crude polysaccharides was conducted according to NY/T 1676-2023 [24] “Determination of Crude Polysaccharide Content in Edible Mushrooms - Spectrophotometric Method” Total sugar content was determined following the national standard GB/T 15672-2009 [25] “Determination of Total Sugar Content in Edible Mushrooms.” Fat content was measured according to the national standard GB 5009.6-2016 [26] “National Food Safety Standard—Determination of Fat in Food.” Ash content was determined following GB 5009.4-2016 [27] “National Food Safety Standard—Determination of Ash in Food.” Protein content was measured according to GB 5009.5-2016 [28] “National Food Safety Standard—Determination of Protein in Food,” using the Kjeldahl method, with a conversion factor of 6.25.

### 2.3. Preparation of PEMFP and Single-Factor Conditions

The extraction method of PEMFP was based on the research of Zhou et al. with modifications [21]. Approximately 10 mg of freeze-dried *Pleurotus eryngii* mushroom feet powder was suspended in different concentrations of NaCl solution at different solid-liquid ratios. Then, extraction at different temperatures for a certain time was assisted by ultrasonics. The PEMFP solution was obtained after centrifugation at 8000 rpm for 15 min. Subsequently, it was dialyzed against a 200 Da MWCO dialysis bag for 24 h to remove salts. The solution was stored at −40 °C and subjected to freeze-drying for 48 h by the vacuum freeze dryer to obtain PEMFP powder. Based on our pre-experiments, the solid-liquid ratio, concentration of NaCl solution, extraction temperature, and extraction time were selected as be four main single factors. Solid-liquid ratio was 1:10, 1:20, 1:30, 1:40, 1:50, and 1:60. Concentration of NaCl solution was 1.5%, 2.5%, 3.5%, 4.5%, and 5.5%. Extraction temperature was 20 °C, 30 °C, 40 °C, 50 °C, and 60 °C. Extraction time was 10 min, 20 min, 30 min, 40 min, and 50 min. A four-factor, three-level Box-Behnken Design (BBD) was employed for RSM optimization. The design included 5 center points for estimating pure error, resulting in a total of 29 experimental runs.

### 2.4. Preparation of PEMFPeps and Single-Factor Conditions

PEMFPeps were prepared by referring to the literature with slight modifications [29]. The lyophilized PEMFP powder was reconstituted in boiling water, and adjusted to different pH by NaOH at different alkaline protease concentration with different enzymatic hydrolysis temperature and enzymatic hydrolysis time. Alkaline protease (from *Streptomyces longus*, ≥100 U/mg) was used to hydrolyze the substrate at 66 °C and pH 9.0, with an enzyme-to-substrate (E:S) ratio of 1:104 for 2.9 h. The determination of the Degree of Hydrolysis (DH) was conducted with reference to the national standard GB 5009.235-2016 [30] “National Food Safety Standard: Determination of Amino Nitrogen in Foods”. The enzymatic reaction was halted by heating at 95 °C for 15 min. The supernatant was collected by centrifugation at 4500 rpm for 20 min, followed by dialysis for 24 h with a 200 Da MWCO dialysis bag. Subsequently, vacuum freeze-drying for 48 h yielded PEMFPeps. Based on our pre-experiments, pH level, alkaline protease concentration, enzymatic hydrolysis temperature, and enzymatic hydrolysis time were selected as be four main single factors. Alkaline protease concentration was 0.5%, 0.75%, 1%, 1.25%, 1.5%, and 1.75%. Enzymatic hydrolysis pH was 6, 7, 8, 9, 10, and 11. Enzymatic hydrolysis time was 1.5 h, 2.0 h, 2.5 h, 3.0 h, 3.5 h, and 4 h. Enzymatic hydrolysis temperature was 35 °C, 45 °C, 55 °C, 65 °C, and 75 °C.

### 2.5. Experimental Design and Statistical Analysis

In the present study, we used Design Expert Version 8.0.6 software as a design and analysis tool to conduct experiments. Regression coefficients, significance of the process variables, conformity of the experimental data to models, and optimal response variables could be obtained by using this software. A four-factor RSM was applied for determining optimal extraction process conditions. The accuracy of the model was investigated by the regression analysis (R^2^). An F-test was conducted to analyze the significance of the model terms. The response surface plots and contour plots were used in combination to show how the process variables affected the response variables. The final quadratic model, including regression coefficients with 95% confidence intervals, analysis of variance (ANOVA) with lack-of-fit test, and residual diagnostics, is presented in the results. The model’s predictive ability was validated externally by conducting experiments at the optimal conditions.

### 2.6. Preparation of SID-PEMFPeps

The method of preparing SID-PEMFPeps was derived from the study of Brodkorb with certain modifications [31]. The simulated digestion in vitro process began with oral digestion, where PEMFPeps were added to 10 mL of simulated saliva fluid, and placed on a shaking incubator at 37 °C for 2 min. The oral-digested samples were mixed with 20 mL of simulated gastric fluid, and the pH was adjusted to 3.0. Pepsin was added to achieve 2000 U/mL in the mixture, and digestion was carried out at 37 °C for 2 h. This was followed by intestinal digestion, where the gastric-digested samples were mixed with 40 mL of simulated intestinal fluid, and the pH was adjusted to 7.0. Pancreatic enzymes were added to achieve the activity level of 100 U/mL in the mixed solution, and digestion was conducted at 37 °C for 2 h. Finally, the digestive fluids were immersed in boiling water for 10 min to deactivate the enzymes and terminate the digestion process. Afterward, the samples were centrifuged at 4500 rpm for 30 min at 4 °C. Once centrifugation was complete, the supernatant was collected and stored at −40 °C. SID-PEMFPeps were obtained by freeze-drying the supernatant in a vacuum freeze dryer for 48 h. The mass distribution of PEMFPeps is provided in Figure S2. Additionally, the LC-MS/MS spectra of PEMFPeps and SID-PEMFPeps, along with the main peptide sequences, are provided in Figure S3, Tables S1 and S2.

The method of preparing SID-PEMFPeps was derived from the study of Brodkorb with certain modifications. The in vitro digestion process began with oral digestion, where PEMFPeps were added to 10 mL of simulated saliva fluid and placed in a shaking incubator at 37 °C for 2 min. After oral digestion, the samples were mixed with 20 mL of simulated gastric fluid, and the pH was adjusted to 3.0. Pepsin (2000 U/mL) was added, and the mixture was incubated at 37 °C for 2 h. Following gastric digestion, intestinal digestion was initiated by mixing the gastric-digested samples with 40 mL of simulated intestinal fluid, and the pH was adjusted to 7.0. Pancreatic enzymes (100 U/mL) were added, and digestion was carried out at 37 °C for 2 h.

### 2.7. Cultivation of PC12 Cells

PC12 cells were obtained from Fuheng Life Sciences (Shanghai, China). The cells were cultured in a medium of 85% RPMI 1640, 5% fetal bovine serum (FBS), 10% horse serum (HS), and 1% penicillin-streptomycin. Cultivation was performed in the CO_2_ incubator at 37 °C with a 5% CO_2_ concentration for 24 h. After culturing until the cell concentration reached 80–90%, the cells were inoculated into 96-well plates to detect cytotoxicity and 6-well plates for other experiments.

### 2.8. Screening of Appropriate SID-PEMFPeps Concentration and Construction of Senescent Cell Model Induced by D-Gal

In this study, the screening of SID-PEMFPeps concentration and the construction of D-Gal induced senescent cell model were based on our previous research [14]. First, appropriate sample concentrations were determined to avoid cell death induced by SID-PEMFPeps. Logarithmic growth phase cells were taken, tryptase (TE) digested, made into a cell suspension, and inoculated at 1 × 105 cells/mL in 96-well plates as the control, the model, and the SID-PEMFPeps group, respectively. After overnight adherence to the culture dishes, PC12 cells in the control and the model groups were treated with RPMI 1640 without FBS and HS for 24 h. In contrast, PC12 cells in the SID-PEMFPeps groups were treated with different concentrations of SID-PEMFPeps (0.25, 0.5, 1, and 2.5 mg/mL) and incubated for 24 h at 37 °C in a 5% CO_2_ incubator to evaluate the cytotoxicity of the samples on PC12 cells. PC12 cells were exposed to different concentrations of D-Gal (2.5, 5, 10, 15, 20, and 25 mg/mL) to determine the appropriate D-Gal concentration for senescence induction. Cell viability was assessed using the CCK-8 assay kit.

### 2.9. RNA Extraction and Transcriptome Analysis

The complete mRNA sequences were enriched from PC12 cells and subjected to random fragmentation in the presence of the fragmentation buffer. The double-stranded cDNA synthesis was then performed using reverse transcriptase and random primers. The sticky ends of the double-stranded cDNA were then repaired to have blunt ends by adding End Repair Mix. Following this, an A base was added to the 3′ ends of the cDNA, and adaptors were ligated. Finally, the resulting products were purified and size-selected. The size-selected fragments were used for PCR amplification, resulting in the generation of the final library.

Differential gene expression analysis was performed using DESeq2, with filtering criteria set at a fold change (FC) of ≥1.7 and a *p*-value < 0.05 to identify differentially expressed genes (DEGs). To account for multiple testing, *p*-values were adjusted using the Benjamini and Hochberg (BH) correction, with a false discovery rate (FDR) threshold of <0.05. Additionally, effect sizes were calculated for all DEGs to assess the magnitude of expression changes. Pathway analysis was performed using Gene Ontology (GO) and Kyoto Encyclopedia of Genes and Genomes (KEGG) databases to identify significantly enriched biological processes and pathways, with pathway selection based on a corrected *p*-value (FDR < 0.05) and enrichment score. Gene–metabolite integration was achieved by correlating DEGs with metabolites identified through metabolomic analysis. Association metrics such as Pearson correlation coefficients and Spearman’s rank correlation were used to evaluate the strength of these relationships.

### 2.10. Untargeted Metabolomic Analysis

Sample analysis was performed using liquid chromatography-tandem mass spectrometry (LC-MS/MS). The chromatographic conditions involved the separation of 3 μL of sample on an HSS T3 column (100 mm × 2.1 mm i.d., 1.8 µm), followed by mass spectrometry detection. The mobile phase A consisted of 95% water and 5% acetonitrile with 0.1% formic acid, while the mobile phase B was composed of 47.5% acetonitrile, 47.5% isopropanol, 5% water, and 0.1% formic acid. The flow rate was set at 0.40 mL/min, and the column temperature was maintained at 40 °C.

Positive and negative ion scanning modes were used in the sample mass spectrometry signal acquisition, with a mass scanning range of 70–1050 *m*/*z*. The sheath gas flow rate was set at 50 psi, the auxiliary gas flow rate at 13 psi, and the auxiliary gas heating temperature at 425 °C. The ionization voltage was set at 3500 V for positive mode and −3500 V for negative mode. The ion transfer tube temperature was maintained at 325 °C, and collision energies were normalized to 20-40-60 V for collision-induced dissociation (CID) cycles. The first-level mass spectrometry resolution was 60,000, and the second-level mass spectrometry resolution was 7500. Data acquisition was performed using Data-Dependent Acquisition (DDA) mode.

MS and MS/MS information were matched against the Metabolomics Databases HMDB (http://www.hmdb.ca/, accessed on 8 July 2023) and Metlin (https://metlin.scripps.edu/, accessed on 8 July 2023) to obtain metabolite information. The R language package “ropls” (Version 1.6.2) was utilized for Principal Component Analysis (PCA) and Orthogonal Partial Least Squares Discriminant Analysis (OPLS-DA) of the preprocessed data matrix. Significant differential metabolites were selected based on Variable Importance in Projection (VIP) values obtained from the OPLS-DA model. Differential metabolites were subjected to metabolic pathway annotation using the KEGG database (https://www.kegg.jp/kegg/pathway.html, accessed on 15 July 2023) to identify the pathways in which these metabolites were involved. Differential metabolites were selected based on a Variable Importance in Projection (VIP) score from the OPLS-DA model >1.5 and a Student’s *t*-test *p*-value < 0.05. False discovery rate (FDR) correction was applied for multiple testing in pathway enrichment analysis.

## 3. Results

### 3.1. Nutritional Composition Results of Pleurotus eryngii and PEMFPeps

The nutritional composition results of *Pleurotus eryngii* and PEMFPeps are as follows:

Protein content: *Pleurotus eryngii*: 13.88 ± 0.188%, PEMFPeps: 46.25 ± 1.864%

Crude polysaccharide content: *Pleurotus eryngii*: 6.30 ± 0.16%, PEMFPeps: 3.11 ± 0.84%

Total sugar content: *Pleurotus eryngii*: 58.16 ± 0.44%, PEMFPeps: 40.71 ± 0.77%

Fat content: *Pleurotus eryngii*: 4.33 ± 0.17%, PEMFPeps: 3.93 ± 0.27%

Ash content: *Pleurotus eryngii*: 5.65 ± 0.33%, PEMFPeps: 3.67 ± 0.88%

### 3.2. Factors Influencing the Extraction Rate of PEMFP

There are many factors affecting the extraction rate of PEMFP. Four main factors, solid-liquid ratio, concentration of NaCl solution, extraction temperature, and extraction time, were investigated to study their influences separately. Single-factor analysis was performed with one factor changed and the others kept unchanged. The concentration of NaCl solution (1.5%, 2.5%, 3.5%, 4.5%, and 5.5%) was investigated, while the other conditions were kept the same (the solid-liquid ratio was 1:40, the extraction temperature was 20 °C, and the extraction time was 20 min). Extraction temperature (20 °C, 30 °C, 40 °C, 50 °C, and 60 °C) was investigated, while the other conditions were kept the same (the solid-liquid ratio was 1:40, the concentration of NaCl solution was 4.5%, and the extraction time was 20 min). Extraction time (10 min, 20 min, 30 min, 40 min, and 50 min) was investigated, while the other conditions were kept the same (the solid-liquid ratio was 1:40, the concentration of NaCl solution was 4.5%, and the extraction temperature was 30 °C). The solid-liquid ratio (1:10, 1:20, 1:30, 1:40, 1:50, and 1:60) was investigated, while the other conditions were kept the same (concentration of NaCl solution was 4.5%, extraction temperature was 30 °C, and extraction time was 30 min).

**Figure 1:**
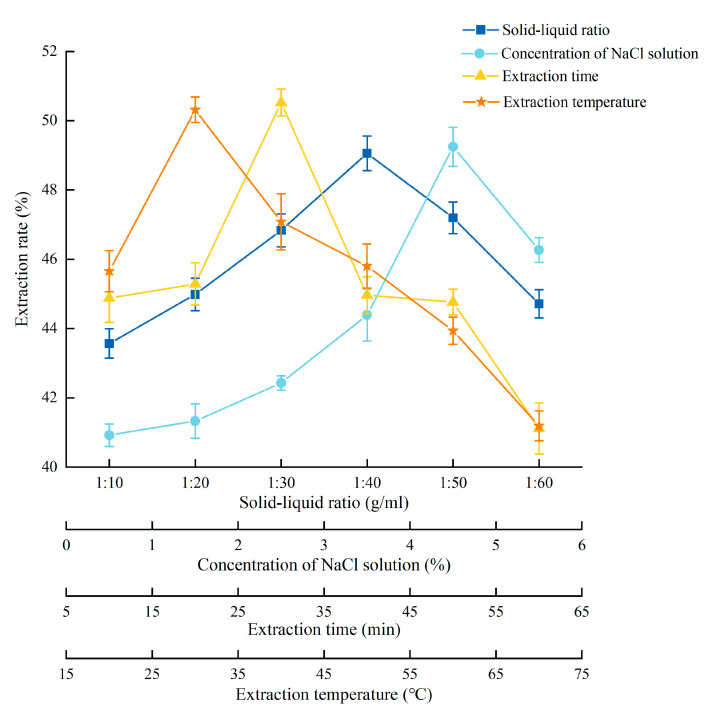
Experimental conditions and results of single-factor analysis of PEMFP.

The detailed experimental conditions and results are shown in Figure 1. As the solid-liquid ratio increased, the dissolved protein gradually increased. The extraction rate became higher and reached its highest when the solid-liquid ratio was 1:40. Then, as the solid-liquid ratio continued to increase, the more other impurities were dissolved, the extraction rate decreased instead. As the concentration of NaCl solution increased, the ion concentration and surface charge of protein gradually increased, and the extraction rate reached the highest when the concentration of NaCl solution was 4.5%. After that, the extraction rate decreased. As the extraction time increased, the extraction rate increased, and the extraction rate reached its highest when the extraction time was 30 min. The extraction rate increased with the extraction temperature increased, and reached the highest when the extraction temperature was 30 °C. When the temperature was higher, the hydrophobic groups would also react, which decreased the extraction rate. Therefore, the center point (all variables were coded as zero) of RSM was 1:45 (solid-liquid ratio), 4% (concentration of NaCl solution), 30 min (extraction time), and 30 °C (extraction temperature).

### 3.3. Optimization of Experimental Conditions by the RSM and the Extraction Rate of PEMFP

An analysis of variance (ANOVA) was conducted and the regression model was summarized in Table 1. By multiple regression analysis, the following second-order polynomial could be obtained as Equation (1).

(1) Y = 55.02 − 2.58A − 1.59B − 1.93C − 0.46D + 3.04AB + 1.35AC − 3.58AD − 1.11BC − 2.95BCD − 8.23A^2^ − 3.20B^2^ − 2.96C^2^ − 4.82D^2^

As can be seen in Table 1, the *F* value of the model was 68.11 while the *p* value was < 0.001, which showed a high significance of the model. For a good accuracy of a model, *R*^2^ must be more than 75% [32]. Thus, the model stated above with a relatively high coefficient of determination value (R^2^ = 0.9855) illustrated that almost all extraction data could be explained by this model. The *p*-value of the simulation error lack-of-fit term is greater than 0.05, indicating that the lack of fit of this model is not significant. This suggests that the equation has a good fit to the experimental data and is highly reliable. The residual analysis shows a mean square of 0.86, indicating a good model fit with no signs of heteroscedasticity or non-normality. Therefore, using this model to predict the influence of the process variables on the protein extraction rate was reasonable and reliable. The linear variable A, B, C, two-variable interaction AB, AD, BD, quadratic variable A^2^, B^2^, C^2^, and D^2^, were statistically very significant because the *p* values were all <0.0001, respectively; two-variable interaction AC and BC had influences for the *p* values were 0.0111 and 0.0308, which were less than 0.05.

The linear and quadratic coefficients of each process variable indicated that the influence of factors was solid-liquid ratio > extraction temperature > extraction time > concentration of NaCl solution.

**Figure 2:**
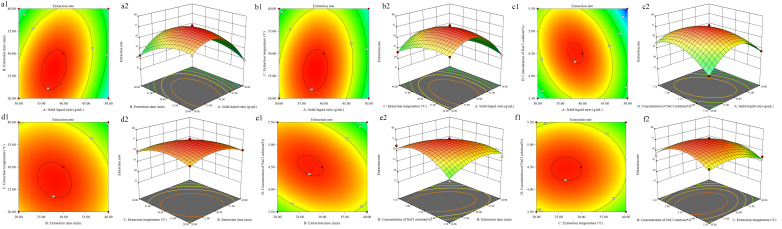
Response surface and contour plots illustrating the interactive effects of solid-liquid ratio (**A**), extraction time (**B**), extraction temperature (**C**), and NaCl concentration (**D**) on the extraction rate of PEMFP. (**a**) A vs. B; (**b**) A vs. C; (**c**) A vs. D; (**d**) B vs. C; (**e**) B vs. D; (**f**) C vs. D. Color representation of extraction rate: red for high, green for low, with gradient indicating intermediate values.

The effects of the process variables and their mutual interactions on the extraction rate could be investigated by the response surface plots and their contour plots. And the interactions between the process variables were significant, while the shape of the contour plots was elliptical. The 3D surfaces and contours plotted in Figure 2a–f displayed the effect of solid-liquid ratio on extraction rate, showing the most obvious in all conditions.

According to the analysis of the response surface, the optimum conditions were obtained: solid-liquid ratio, 1:45.66 (g/mL); extraction time, 32.05 min; extraction temperature, 27.76 °C; concentration of NaCl solution, 4.18%. Under the above conditions, the estimated value for the extraction rate of PEMFP was obtained, which was 51.26%. Considering the practical operability, the actual experiment conditions were adjusted to be: c. After three parallel experiments, the mean value of the extraction rate of PEMFP was 51.31 ± 3.00%, which was close to the estimated value. By comparing the actual experimental and predicted results, the model had a great agreement with the predictive values and could be verified. Therefore, the optimal extraction conditions obtained by RSM were accurate, reliable, and efficient. To mitigate overfitting concerns, the model’s robustness was validated by performing three additional experimental runs at conditions slightly perturbed from the reported optimum. The results confirmed the predictive reliability of the model.

### 3.4. Factors Influencing the Enzymatic Hydrolysis of PEMFPeps

There are many factors affecting the enzymatic hydrolysis rate of peptides. Four main factors, pH level, alkaline protease concentration, enzymatic hydrolysis temperature, and enzymatic hydrolysis time, were investigated to separately study their influences. Single-factor analysis was performed with one factor changed and the others kept unchanged. The pH (6, 7, 8, 9, 10, and 11) was investigated, while the other conditions were kept the same (alkaline protease concentration was 1%, enzymatic hydrolysis temperature was 55 °C, and enzymatic hydrolysis time was 3.0 h). Alkaline protease concentration (0.5%, 0.75%, 1%, 1.25%, 1.5%, and 1.75%) was investigated, while the other conditions were kept the same (pH was 9, enzymatic hydrolysis temperature was 55 °C, and enzymatic hydrolysis time was 3.0 h). Enzymatic hydrolysis time (1.5 h, 2.0 h, 2.5 h, 3.0 h, 3.5 h, and 4.0 h) was investigated, while the other conditions were kept the same (pH was 9, alkaline protease concentration was 1%, and enzymatic hydrolysis temperature was 3.0 h). Enzymatic hydrolysis temperature (35 °C, 45 °C, 55 °C, 65 °C, 75 °C, and 85 °C) was investigated, while the other conditions were kept the same (pH was 9, alkaline protease concentration was 1%, and enzymatic hydrolysis time was 3.0 h).

**Figure 3:**
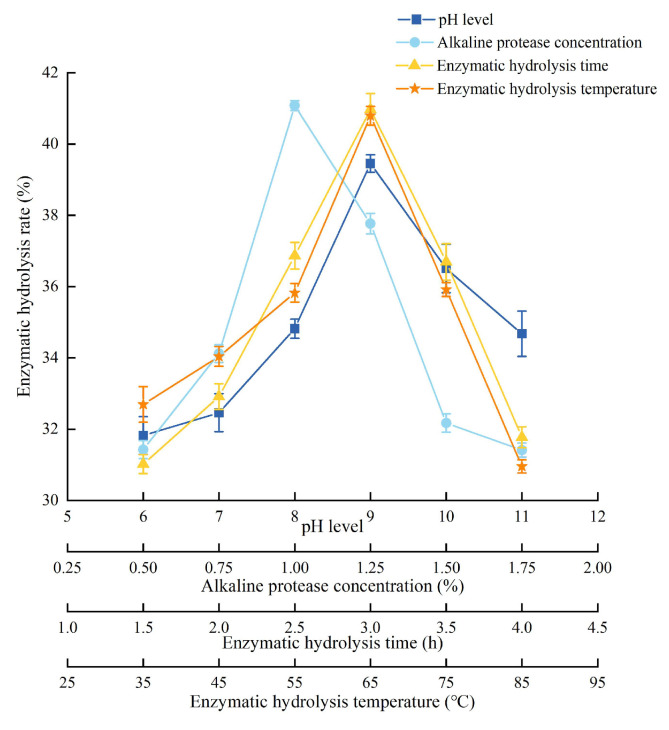
Experimental conditions and results of single-factor analysis of PEMFPeps.

The detailed experimental conditions and results were shown in Figure 3. The enzymatic hydrolysis rate became higher with the pH level increasing, and reached the highest when the pH was 9. And then, the enzymatic hydrolysis rate decreased instead. It could be speculated that the activity of alkaline protease reached the highest at pH 9. The enzymatic hydrolysis rate increased rapidly with the increase in alkaline protease concentration, reached a peak at 1%, and then declined gradually. The enzymatic hydrolysis rate increased as the enzymatic hydrolysis time increased and reached the highest level at 3.0 h. Moreover, the activity of alkaline protease reached saturation. After that, the enzymatic hydrolysis rate decreased. The variation trend of the enzymatic hydrolysis temperature was similar to the enzymatic hydrolysis time. The enzyme activity was the highest when the temperature was 65 °C. Therefore, center point (all variables were coded as zero) of RSM was 9 (pH level), 1% (alkaline protease concentration), 3.0 h (enzymatic hydrolysis time), and 65 °C (enzymatic hydrolysis temperature).

### 3.5. Optimization of Experimental Conditions by the RSM and the Hydrolysis Rate of PEMFPeps

The optimization of experimental conditions by the RSM was similar to Section 3.2. An analysis of variance (ANOVA) was conducted and the regression model was summarized in Table 2. By multiple regression analysis, the following second-order polynomial could be obtained as Equation (2).

(2) Y = 47.40 − 0.32A − 0.002B − 0.34C + 0.52D + 0.37AB + 0.41AC + 0.40AD − 0.22BC + 1.08BD − 0.78CD − 83.15A^2^ − 2.34B^2^ − 1.43C^2^ − 2.54D^2^

As can be seen in Table 2, the *F* value of the model was 473.43 while the *p* value was <0.001, which showed a high significance of the model. For a good accuracy of a model, R^2^ must be more than 75% [32]. Thus, the model stated above with a relatively high coefficient of determination value (R^2^ = 0.9979) illustrated that almost all hydrolysis data could be explained by this model. The *p*-value of the simulation error lack-of-fit term is greater than 0.05, indicating that the lack of fit of this model is not significant. This suggests that the equation has a good fit to the experimental data and is highly reliable. The residual analysis shows a mean square of 0.018, indicating a good model fit with no signs of heteroscedasticity or non-normality. Therefore, using this model to predict the influence of the process variables on the peptides hydrolysis rate was reasonable and reliable. The linear variable A, B, D, two-variable interaction AB, AD, BC, BD, and CD, quadratic variables A^2^, B^2^, C^2^ and D^2^, were statistically very significant because the *p* value were all <0.0001, respectively; two-variable interaction AC had significant influence on the hydrolysis rate of PEMFPeps for the *p* value was 0.0061, which were less than 0.01.

The linear and quadratic coefficients of each process variable indicated that the influence of factors was alkaline protease concentration > enzymatic hydrolysis temperature > enzymatic hydrolysis time > pH level.

**Figure 4:**
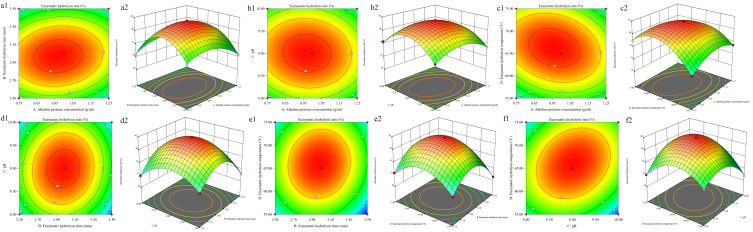
Response surface and contour plots showing the interactive effects of alkaline protease concentration (**A**), enzymatic hydrolysis time (**B**), pH (**C**), and hydrolysis temperature (**D**) on the hydrolysis rate of PEMFPeps. Variable combinations: (**a**) A vs. B; (**b**) A vs. C; (**c**) A vs. D; (**d**) B vs. C; (**e**) B vs. D; (**f**) C vs. D. Color representation of enzymatic hydrolysis rate: red for high, green for low, with gradient indicating intermediate values.

The effects of the process variables and their mutual interactions on the hydrolysis rate can be investigated by the response surface plots and their contour plots. And the interactions between the process variables are significant, while the shape of the contour plots is elliptical. The 3D surfaces and contours plotted in Figure 4a–f displayed the effect of alkaline protease concentration on hydrolysis rate showing the most obvious in all conditions.

According to the analysis of the response surface, the optimum conditions were obtained: alkaline protease concentration 0.96%, enzymatic hydrolysis time 2.98 h, enzymatic hydrolysis temperature 66.3 °C, and pH level 9.03. Under the above conditions, the estimated value for the hydrolysis rate of PEMFPeps was obtained, which was 48.47%. Considering the practical operability, the actual experiment conditions were adjusted to be: alkaline protease concentration 0.96%, enzymatic hydrolysis time 2.9 h, enzymatic hydrolysis temperature 66 °C, and pH level 9. After three parallel experiments, the mean value of the enzymatic hydrolysis rate of PEMFPeps was 48.71 ± 0.17%, which was close to the estimated value. By comparing the actual experimental and predicted content, the model had a great agreement with the predictive values and could be verified. Therefore, the optimal hydrolysis conditions obtained by RSM were accurate, reliable, and efficient.

### 3.6. Determination of Appropriate SID-PEMFPeps Concentration and Construction of Senescent Cell Model Induced by D-Gal

In our previous study [14], we determined that a concentration of 20 mg/mL of D-galactose effectively induces senescence in PC12 cells. To evaluate the effects of SID-PEMFPeps on D-gal-induced senescence, we first assessed its cytotoxicity to ensure that the treatment would not cause cellular damage. SID-PEMFPeps at concentrations of 0.25, 0.5, and 1 mg/mL did not affect cell viability, while 2.5 mg/mL induced significant cytotoxicity. GSH, used as a positive control, did not inhibit cell viability at any concentration. Based on these findings, 0.25, 0.5, and 1.0 mg/mL concentrations were selected for further experiments. SID-PEMFPeps treatment significantly reduced ROS generation by 51.53% (*p* < 0.05) and increased antioxidant enzyme activities, including SOD, GSH-Px, and CAT. Additionally, MDA levels were reduced by 27.11% (*p* < 0.05). The percentage of SA-β-gal-positive cells was significantly lower in SID-PEMFPeps-treated groups compared to the model group. The high-dose SID-PEMFPeps group showed a 39.86% reduction in SA-β-gal-positive cells, indicating a significant decrease in oxidative stress-induced cellular senescence. These results suggest that SID-PEMFPeps alleviates cellular senescence through its antioxidant and anti-aging effects.

### 3.7. Transcriptomic Analysis

#### 3.7.1. Transcriptome Sequencing

Based on our previous experiment results, the SID-PEMFPeps-H group exhibited the most effective protective activity [14]. Therefore, the SID-PEMFPeps-H group was selected for RNA-seq analysis with the control and model groups. The raw reads obtained from the cDNA libraries constructed for the control, model, and SID-PEMFPeps-H groups were 50, 552, 563, 48, 438, 649, and 50, 485, 514, respectively. As shown in Supplementary Materials Table S3, the Q20 and Q30 values were above 94%, indicating that all samples had high sequencing quality, low error rates, and that the sequencing platform was stable and reliable. Each sample had more than 40 million clean reads, making the data suitable for subsequent expression analysis and different expression analysis. The GC content was stable with no significant deviations, ruling out contamination or systematic bias. There was little variation between groups, and there were no significant differences in sequencing quality among the control, model, and SID-PEMFPeps-H groups, indicating that the experimental design was sound.

**Figure 5:**
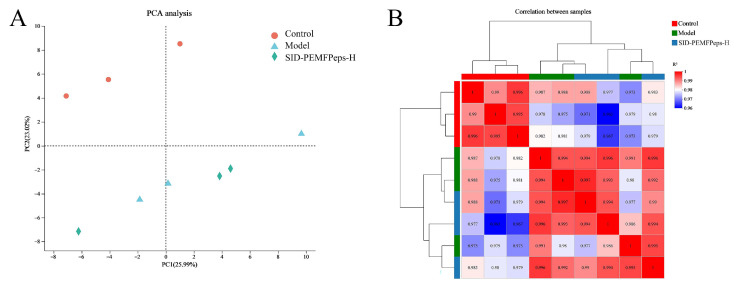
PCA plot (**A**) of transcriptome sequencing and correlation analysis of different groups (**B**). The horizontal coordinate indicates the contribution of principal component 1 (PC1) to distinguish the samples, and the vertical coordinate indicates the contribution of principal component 2 (PC2) to distinguish the samples.

To evaluate the overall quality and consistency of the RNA-Seq data, principal component analysis (PCA) and sample correlation analysis were performed. As shown in Figure 5A, PCA revealed clear separation among the control, model, and SID-PEMFpeps-H groups at the transcriptomic level, indicating significant differences in gene expression among the treatment groups. Control samples clustered tightly in the upper left quadrant, while model group samples were distributed in the lower left quadrant, suggesting that the aging model condition induced notable transcriptomic alterations. In contrast, the SID-PEMFpeps-H samples formed an independent cluster located between the control and model groups, implying that the SID-PEMFPeps-H treatment could partially reverse or modulate the model-induced changes in gene expression.

Correlation analysis further confirmed the consistency of the data (Figure 5B). Pearson correlation coefficients among samples within each group were high (r > 0.95), indicating good intra-group reproducibility. In contrast, inter-group correlations, particularly between the control and model groups, were significantly lower (r < 0.8), which further supported the presence of substantial transcriptomic differences among the groups. These results collectively demonstrated the high quality of the sequencing data and the existence of distinct biological differences under different experimental conditions.

#### 3.7.2. Transcriptomic Profiling Reveals the Restorative Effect of SID-PEMFPeps-H on Global Gene Expression

Volcano plots were employed to visualize differentially expressed genes (DEGs), with the horizontal coordinate representing the log2-transformed fold change and the vertical coordinate indicating the -log10-transformed adjusted *p*-value (adj.P.Val).

**Figure 6:**
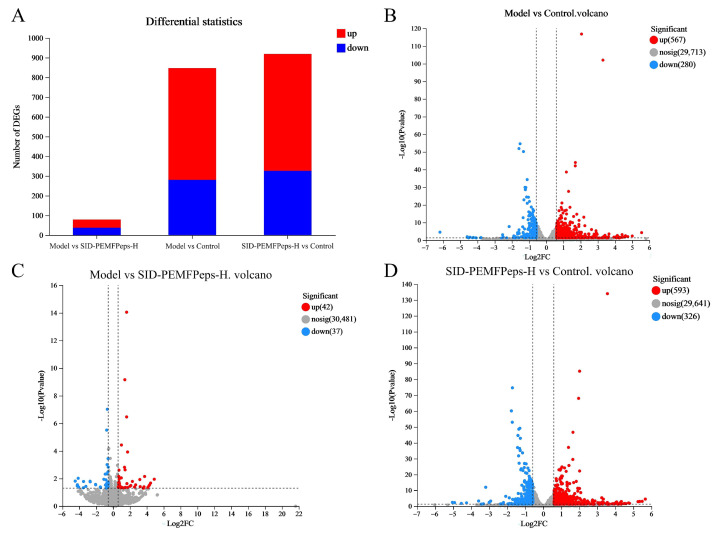
Gene expression analysis. The statistical plot of the differences in expression (**A**), red represented upregulation and blue represented downregulation. Volcano plots of significant DEGs in the model vs. control group (**B**), the model vs. SID-PEMFPeps-H group (**C**), and the SID-PEMFPeps-H vs. control group (**D**). Red dots indicated upregulation of gene expression; blue dots indicated downregulation of gene expression; gray dots indicated non-significantly different genes, and dots closer to the two sides and the top were more significant.

Striking differences in transcriptional landscapes were observed among the groups. The comparison between the Model and Control groups revealed extensive alterations, identifying 847 DEGs (fold-change > 2, *p* < 0.05), of which 567 were upregulated and 280 were downregulated (Figure 6A,B). This widespread dysregulation underscores the profound impact of D-galactose induction on global gene expression, successfully establishing a senescence model.

Critically, the comparison between the Model and SID-PEMFPeps-H groups exhibited a markedly reduced number of DEGs—only 79 in total (42 upregulated, 37 downregulated) (Figure 6A,C). This minimal disparity indicates that the gene expression profile of the SID-PEMFPeps-H treated cells closely resembles that of the Model group. However, far from being insignificant, this near-normalization powerfully suggests that the treatment effectively counteracts the D-galactose-induced transcriptional dysregulation, restoring the expression of a vast majority of genes towards a state akin to the untreated senescent model, but with a critical functional difference as explored below.

Conversely, the comparison between SID-PEMFPeps-H and Control groups also showed a high number of DEGs (919 total; 593 upregulated, 326 downregulated) (Figure 6A,D), indicating that the treatment itself induces substantial transcriptional changes.

Interpretively, these patterns collectively depict a restorative mechanism. The stark contrast between the extensive Model vs. Control DEGs and the minimal Model vs. SID-PEMFPeps-H DEGs demonstrates that SID-PEMFPeps-H does not merely impose a new transcriptional state. Instead, it largely reverses the senescence-associated gene expression signature induced by D-galactose. The high number of DEGs in SID-PEMFPeps-H vs. Control likely represents a composite of this reversal effect and potential pro-homeostatic signals activated by the treatment. This transcriptional restoration provides a robust foundation for the observed phenotypic rescue and guides subsequent pathway analysis to uncover the specific processes being modulated.

#### 3.7.3. Combined KEGG and GO Enrichment Analyses Reveal Anti-Aging Mechanisms of SID-PEMFPeps-H

**Figure 7:**
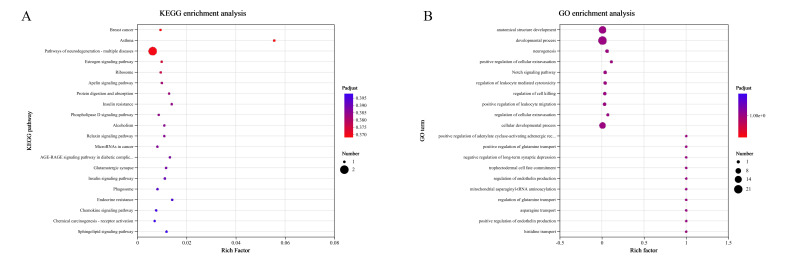
Enrichment analysis of differentially expressed genes between the model vs. control group and the model vs. SID-PEMFPeps-H group. (**A**) KEGG pathway enrichment analysis showing the top 20 significantly enriched pathways ranked by padjust; (**B**) GO biological process enrichment analysis showing the top 20 enriched GO terms.

To explore the molecular mechanisms underlying the aging phenotype and the potential anti-aging effects of SID-PEMFPeps-H, KEGG (Figure 7A) and GO (Figure 7B) enrichment analyses were conducted separately for the differentially expressed genes obtained from the model vs. control group and the model vs. SID-PEMFPeps-H group. The KEGG enrichment analysis (Figure 7A) highlighted the pathways significantly enriched with DEGs. Each dot represented a pathway, with the size of the dot correlating to the number of DEGs involved in that pathway, and the color intensity indicating the level of statistical significance (Padjust), with redder colors representing more significant enrichment. The GO enrichment analysis (Figure 7B) focused on the functional categorization of DEGs into three main categories: biological processes, molecular functions, and cellular components. Similarly to the KEGG analysis, the size of the dot indicated the number of DEGs, and the color intensity reflected the significance level (Padj).

KEGG analysis revealed 20 significantly enriched pathways, with key pathways highlighted according to padjust values, Rich factors, and gene counts, including Breast cancer (cancer-related) [33], Asthma (oxidative stress-related) [34], Pathways of neurodegeneration-multiple diseases (related to neurodegenerative diseases) [35], estrogen signaling pathway (involved in metabolic regulation) [36], and ribosome (associated with protein synthesis) [37]. A summary of the main KEGG pathways enriched in the transcriptomic analysis is provided in Supplementary Materials Table S4. These findings suggested that SID-PEMFPeps-H might exert its effects through mechanisms such as oxidative stress modulation, immune regulation, and metabolic activity enhancement. Notably, most of these pathways were closely related to aging, neurodegeneration, and metabolic dysfunction.

GO enrichment analysis further revealed that SID-PEMFPeps-H extensively affected the development of anatomical structures at the levels of organs, tissues, and cells. “Anatomical structure development”, “developmental process”, and “cellular developmental process” were significantly enriched, indicating that SID-PEMFPeps-H might influence developmental and structural aspects of organisms. Notably, glutamatergic synapse and anatomical structure development were identified as common enriched pathways in both model vs. control and model vs. SID-PEMFPeps-H comparisons. The former plays a central role in excitatory neurotransmission and synaptic plasticity in the central nervous system, while the latter broadly refers to the formation and remodeling of cellular and tissue structures. Previous studies have shown that D-galactose induces age-related synaptic dysfunction through impaired glutamatergic signaling and reduced expression of synaptic markers in the hippocampus and prefrontal cortex, ultimately disrupting synaptic structure and function [38, 39]. Moreover, interventions such as antioxidants have been reported to mitigate D-galactose-induced neuronal damage by enhancing hippocampal neurogenesis and restoring synaptic plasticity [40]. The intersection of glutamatergic signaling and structural development in our analysis suggested that synaptic structure and function might be compromised during D-galactose-induced cellular aging, and that SID-PEMFPeps-H might exert neuroprotective effects by partially restoring synaptic integrity and activity.

### 3.8. Metabolome Analysis

#### 3.8.1. PCA Plots, OPLS-DA Plots, VIP Score and Potential Metabolite Biomarkers

**Figure 8:**
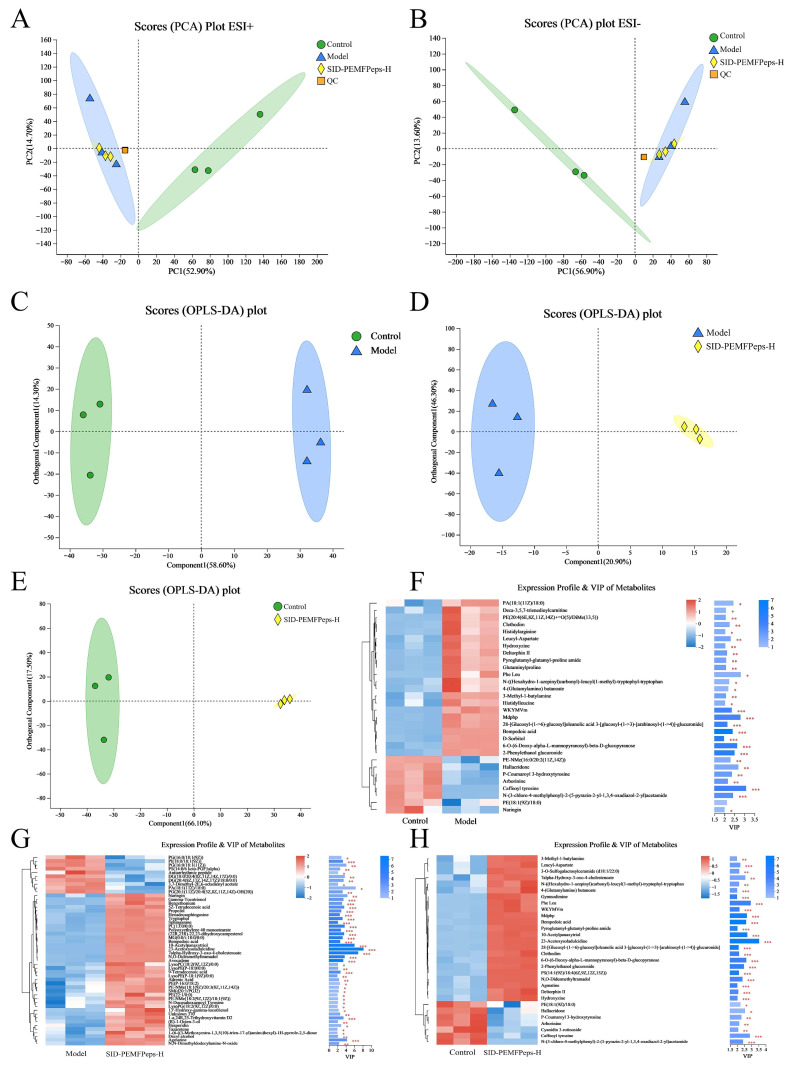
Metabolome analysis. PCA plots of positive (**A**) and negative (**B**) ions. OPLS-DA plots of the model vs. control group (**C**), the model vs. SID-PEMFPeps-H group (**D**) and the SID-PEMFPeps-H vs. control group (**E**). VIP score analyses of the model vs. control group (**F**), the model vs. SID-PEMFPeps-H group (**G**) and the SID-PEMFPeps-H vs. control group (**H**). Statistical significance was determined by Student *t*-test. * *p* < 0.05, ** *p* < 0.01, *** *p* < 0.001.

The metabolic changes in the model vs. control group, the model vs. SID-PEMFPeps-H group, and the SID-PEMFPeps-H vs. control group were analyzed, leading to the detection of a total of 1776 annotated metabolites (993 in ESI+ and 783 in ESI- mode). PCA demonstrated significant differences in all groups in both positive and negative modes, as illustrated in Figure 8A,B. Notably, there were distinct differences between the annotated metabolites of the three groups, indicating that the metabolism of PC12 cells was altered by the process of modeling with D-Gal and adding SID-PEMFPeps. 466, 92 and 573 annotated metabolites were selected between the model vs. control group, the model vs. SID-PEMFPeps-H group and the SID-PEMFPeps-H vs. control group, respectively.

Metabolite clustering was validated by constructing OPLS-DA and S-plot plots and further mined the potential biomarkers after SID-PEMFPeps intervention. The first principal component explained 58.60%, 20.90% and 66.10% of the total variance of the comparison of the model vs. control group, the SID-PEMFPeps-H vs. model group and the SID-PEMFPeps-H vs. control group, with corresponding Q2 values were 0.957, 0.700 and 0.980, respectively, which indicated the reliability of the proposed model (Figure 8C–E).

It has been shown that tryptophol is a derivative of tryptophan, involved in tryptophan metabolism, and has been identified as a potential biomarker associated with aging [41], Arborinine, adrenic acid, and caffeoyl tyrosine have been demonstrated to possess anti-inflammatory and anti-aging properties [42, 43, 44]. Bempedoic acid extends the human lifespan by lowering the risk of coronary heart disease through lipid-lowering [45, 46]. 27 metabolites had VIP scores > 2.0 in the model vs. control group (Figure 8F), including caffeoyl tyrosine (VIP = 3.07), hallacridone (VIP = 2.68), and bempedoic acid (VIP = 2.39). There were 45 metabolites with VIP scores > 2.0 in the model vs. SID-PEMFPeps-H group (Figure 8G), consisting of 7α-hydroxy-3-oxo-4-cholestenoic (VIP = 6.84), tryptophol (VIP = 3.14), and adrenic acid (VIP = 2.31). 26 metabolites in the SID-PEMFPeps-H vs. control groups had VIP scores > 2.0 (Figure 8H), including 23-acetoxysoladulcidine (VIP = 3.53), PheLeu (VIP = 2.93), and arborinine (VIP = 2.12).

**Figure 9:**
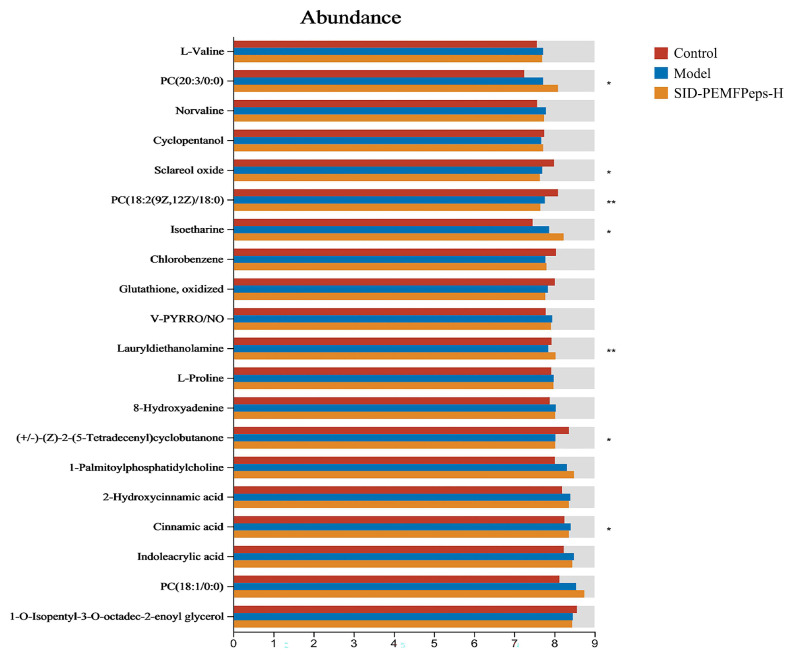
Selected potential metabolite biomarkers in control, model, and SID-PEMFPeps-H groups. Statistical significance was determined by Student *t*-test. * *p* < 0.05, ** *p* < 0.01.

According to the analysis conducted with OPLS-DA and S-Plot, potential biomarkers, including L-valine, norvaline, L-proline, 8-hydroxyadenine, 2-hydroxycinnamic acid, cinnamic acid, and indoleacrylic acid, exhibited remarkable increases in the model vs. control group (*p* < 0.05). In contrast, cyclopentanol, chlorobenzene and lauryldiethanolamine showed a significant decline. After treatment with SID-PEMFPeps, a reversal of the trend of each potential biomarker was observed (Figure 9). Thus, it was concluded that the SID-PEMFPeps could protect neurons from oxidative stress-induced cell death and achieve anti-aging effects by modulating anti-inflammatory factors and anti-apoptotic factors such as arborinine, adrenic acid, caffeoyl tyrosine, and bempedoic acid.

#### 3.8.2. Kyoto Encyclopedia of Genes and Genomes (KEGG) Enrichment Analysis

**Figure 10:**
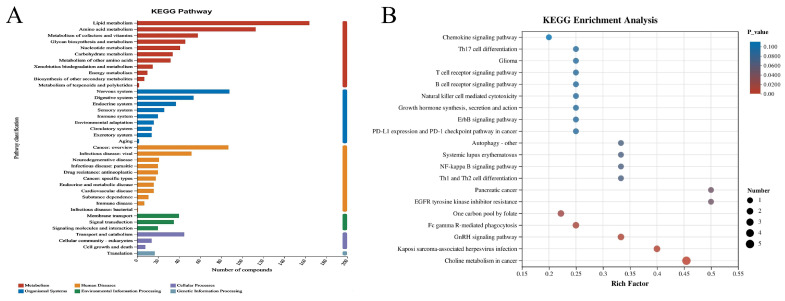
KEGG pathway map (**A**), the horizontal coordinate was the number of compounds under the pathway and the vertical coordinate was the name of the KEGG pathway. KEGG enrichment bubble chart (**B**). The size of the bubbles represented the number of metabolites in each KEGG pathway. The color of the bubbles corresponded to the magnitude of the different enrichment significance *p* values.

Multiple differentially expressed metabolites were mapped to and classified into several major biological categories in the KEGG database (Figure 10A). In particular, pathways related to metabolism were predominant, accounting for a significant proportion of the total. Bubble plot analysis (Figure 10B) further demonstrated that the chemokine signaling pathway, Th17 cell differentiation, and glutamatergic synapse were among the highly enriched pathways, characterized by high Rich Factors and low *p* values. This suggested their potential involvement in immune modulation and neurotransmission. Detailed KEGG tabular data (Supplementary materials Table S5) supported these findings, revealing that top pathways like autophagy, purine metabolism, ABC transporters, and arachidonic acid metabolism played significant roles in cellular catabolism, nucleotide turnover, and inflammation regulation. Additionally, several disease-related pathways, such as those involved in Kaposi sarcoma-associated herpesvirus infection, Parkinson’s disease, and cancer, showed notable metabolite involvement. This indicated a broader pathophysiological relevance.

These results suggested that the intervention influenced a wide range of metabolic networks, particularly those related to lipid metabolism, amino acid turnover, and immune and nervous system signaling pathways. These metabolic changes might be the basis for the observed biological effects.

### 3.9. Integrated Multi-Omics Analysis Elucidates a Coherent Mechanism of Cellular Rejuvenation

To obtain a systems-level understanding of the anti-aging mechanism, we integrated the transcriptomic and metabolomic datasets. This unified analysis revealed a compelling, synergistic picture of how SID-PEMFPeps-H counteracts D-galactose-induced senescence.

#### 3.9.1. Synergistic Regulation of Central Carbon Metabolism

**Figure 11:**
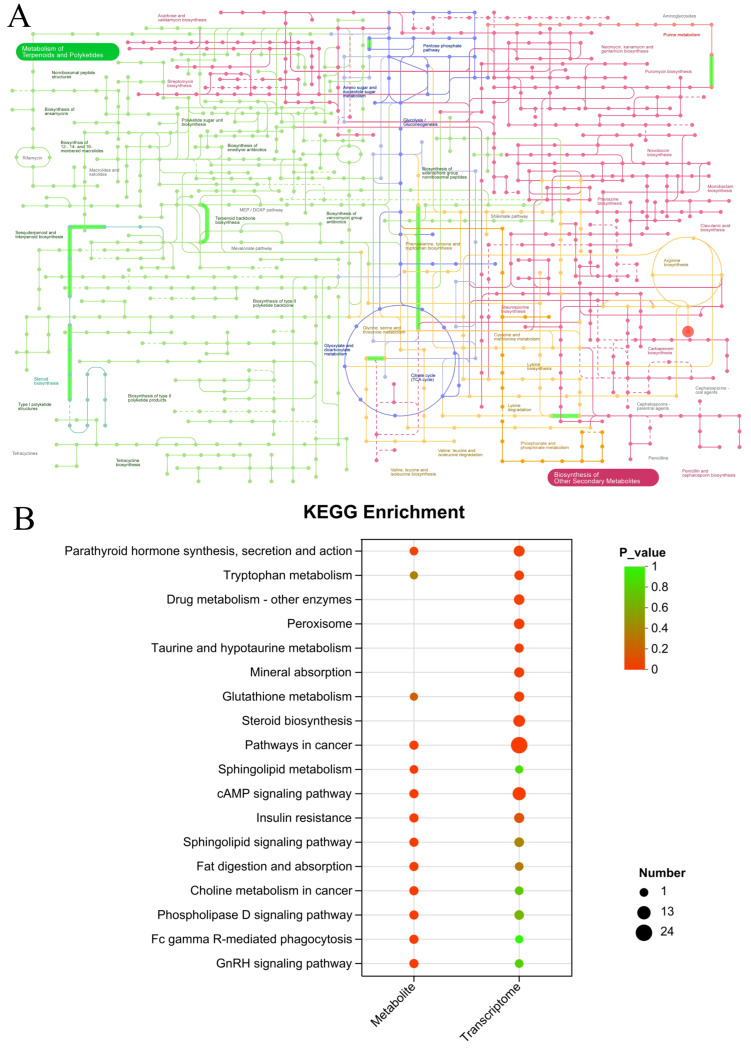
Integrated pathway analysis. (**A**) iPath map of differential genes and metabolites. (**B**) KEGG enrichment of co-regulated pathways from transcriptomics and metabolomics.

The iPath analysis highlighted a concerted modulation of core metabolic pathways, including the pentose phosphate pathway (PPP), glycolysis/gluconeogenesis, the TCA cycle, and amino sugar/nucleotide sugar metabolism (Figure 11A). The coordinated regulation of the PPP and TCA cycle is particularly insightful. It suggests that SID-PEMFPeps-H alleviates oxidative stress through a dual mechanism: enhancing NADPH production via the PPP to bolster antioxidant defenses, and restoring mitochondrial energy metabolism via the TCA cycle to overcome the bioenergetic deficit characteristic of aging [47, 48]. Concurrently, the alterations in amino sugar and nucleotide sugar metabolism point to improved support for cell membrane integrity and glycosylation processes, which are crucial for cellular repair and signaling [49, 50].

#### 3.9.2. Convergence on Key Signaling Pathways and Functional Recovery

Joint KEGG enrichment analysis identified five pivotal pathways shared between the transcriptomic and metabolomic datasets: cAMP signaling, pathways in cancer, non-small cell lung cancer, glutamatergic synapse, and parathyroid hormone synthesis, secretion and action (Figure 11B). Among these, the cAMP signaling pathway and the glutamatergic synapse stand out as central hubs for neuroprotection and metabolic regulation. This convergence indicates that the therapeutic effects of SID-PEMFPeps-H are mediated through the modulation of these critical signaling cascades, which govern everything from neuronal excitability and synaptic plasticity to systemic energy homeostasis.

#### 3.9.3. Reversal of Key Aging-Associated Gene Expression

The power of the integrated approach is further evidenced by the reversal of specific aging-associated gene expressions. In the model group, we observed significant upregulation of stress-responsive genes (e.g., *Arap3* [51], *Grin2d* [52]) and downregulation of neuroprotective and developmental genes (e.g., *Npy1r* [53], *Wnt4*, *Mafb* [54]). Strikingly, SID-PEMFPeps-H treatment consistently reversed these aberrant expression trends, normalizing the levels of these critical regulators.

This gene expression reversal, when viewed in concert with the metabolic and pathway alterations, constructs a coherent mechanistic narrative: SID-PEMFPeps-H rectifies the D-galactose-induced bioenergetic and redox imbalance (evidenced by metabolic pathway changes). This restored cellular fitness, in turn, enables the reactivation of vital neuroprotective signaling (e.g., cAMP, NPY) and the repression of detrimental processes (e.g., excitotoxicity via *GluN2D*, inflammatory remodeling via *Arap3*). The collective effect is a functional restoration of synaptic integrity, a reduction in inflammatory stress, and a promotion of cellular repair, ultimately culminating in the observed attenuation of the senescent phenotype.

#### 3.9.4. Potential Gene–Metabolite Associations and Future Validation

Although quantitative gene–metabolite correlation analysis was not conducted in this study, the integrated enrichment of oxidative stress–related pathways (such as the pentose phosphate pathway, TCA cycle, and glutamatergic synapse) and the coordinated changes in key genes (*Arap3*, *Grin2d*, *Npy1r*) and metabolites (L-valine, norvaline, and L-proline) strongly suggest functional associations between the transcriptomic and metabolomic layers. These findings indicate a coherent regulatory network in which SID-PEMFPeps-H alleviates oxidative and metabolic stress, thereby restoring neuronal and cellular homeostasis.

In future work, targeted validation such as qPCR for key genes and targeted metabolite quantification will be performed to further confirm these predicted associations and strengthen the mechanistic insights of this study.

## 4. Discussion

This study successfully established an optimized pipeline for producing bioactive peptides from *Pleurotus eryngii* by-products and provided a systems-level understanding of their anti-aging mechanism using integrated transcriptomics and metabolomics. Our discussion will first contextualize the optimization process, then delve into the biological findings, comparing them with existing literature, and finally address the study’s implications and limitations.

### 4.1. Production and Optimization Efficiency

The RSM-optimized processes for protein extraction and enzymatic hydrolysis yielded high efficiencies of (51.31 ± 3.00)% and (48.71 ± 0.17)%, respectively. These values are notably higher than those reported for proteins extracted from other edible fungi, such as *Hericium erinaceus* mycelium (27.2%) [55] and *Coprinus comatus* (18.49%) [56], and are also comparable to plant-derived proteins such as pea or soybean isolates, which typically yield between 38–50% depending on pH and enzyme treatment [57]. The robustness of our models, confirmed by validation experiments, ensures the reproducibility of peptide production, which is crucial for future applications.

### 4.2. Anti-Senescence Activity and Underlying Mechanisms

The potent anti-senescence effect of SID-PEMFPeps in D-gal-induced PC12 cells is consistent with the antioxidant and mitochondrial rationale. Our multi-omics data sig-nificantly expand upon our previous report on the TLR4/NF-κB/MAPK pathway [14], revealing a more comprehensive network. The reversal of D-gal-induced transcriptomic and metabolic alterations by SID-PEMFPeps highlights its restorative, rather than merely protective, property.

The enrichment of pathways like glutamatergic synapse, cAMP signaling, and central carbon metabolism (PPP, TCA cycle) points to a multi-targeted mechanism. While our integrated analysis strongly associates these pathway modulations with the anti-aging phenotype, future studies employing pathway-specific inhibitors (e.g., PKA inhibitors for cAMP signaling) or genetic interventions (e.g., TLR4 knockdown) are necessary to establish causality.

The identification of specific genes (*Arap3*, *Grin2d*, *Npy1r*) and metabolites (L-valine, norvaline) provides molecular targets. The role of *Npy1r* in neuroprotection [53] and its reversal by our treatment is particularly intriguing. Compared to anti-aging peptides derived from *Ganoderma lucidum* [10], which primarily target inflammation, SID-PEMFPeps appear to have a broader impact on energy metabolism and synaptic function, potentially delineating the unique contribution of the *Pleurotus eryngii* matrix.

### 4.3. Limitations and Future Perspectives

It is important to acknowledge the limitations of this study. Firstly, the findings are based on an in vitro cell model. Future work must include validation in an in vivo model, such as D-galactose-induced aging mice, assessing behavioral outcomes and tissue-specific markers. Secondly, while we characterized the peptide profiles, the specific peptide sequence (s) responsible for the observed bioactivity remain to be identified and synthesized for confirmation. In future studies, we will also include additional functional markers such as ΔΨm, ATP, NADPH/NADP+, apoptosis markers (e.g., Annexin V), and neurite morphology to further strengthen our mechanistic conclusions. Lastly, the safety, long-term stability, and oral bioavailability of these peptides require thorough investigation before any translational application in functional foods or supplements.

## 5. Conclusions

This study successfully establishes an efficient pipeline for the valorization of *Pleurotus eryngii* processing by-products. Through RSM optimization, we achieved a high extraction yield of protein (PEMFP, 51.31 ± 3.00%) and subsequently generated bioactive peptides (PEMFPeps) with a notable degree of hydrolysis (48.71 ± 0.17%), providing a sustainable strategy for resource utilization.

Moving beyond process optimization, our work delivers unprecedented insights into the anti-aging mechanisms of the simulated digests (SID-PEMFPeps). By employing an integrated transcriptomic and metabolomic approach, we demonstrated that SID-PEMFPeps-H alleviates D-galactose-induced senescence through a multi-faceted mechanism that orchestrates global transcriptional and metabolic reprogramming. This systems-level perspective significantly expands upon our previous finding of TLR4/NF-κB/MAPK pathway involvement [14], revealing a more comprehensive network of action.

Key findings from the multi-omics integration include:

1. Energetic and Redox Restoration: SID-PEMFPeps-H rectifies the aging-associated bioenergetic deficit and oxidative stress by coordinately regulating central carbon metabolism, including the pentose phosphate pathway, glycolysis/gluconeogenesis, and the TCA cycle, thereby enhancing NADPH production and mitochondrial energy metabolism;
2. Neuronal and Structural Repair: The intervention significantly impacted pathways crucial for brain health, such as the glutamatergic synapse and those involved in anatomical structure development, indicating a restorative effect on neuronal function and synaptic integrity;
3. Pleiotropic Signaling Modulation: Joint pathway analysis highlighted the compound’s influence on key signaling hubs, including the cAMP signaling pathway, underscoring its ability to modulate critical processes in signal transduction and neuroendocrine function;
4. Gene Expression Reversal: The treatment effectively reversed the aberrant expression of key aging-associated genes implicated in cytoskeletal remodeling (*Arap3*), excitotoxicity (*Grin2d*), and neuroprotection (*Npy1r*, *Wnt4*, *Mafb*), providing molecular-level evidence of cellular homeostasis restoration.

Crucially, by employing an integrated transcriptomic and metabolomic approach, we demonstrated that the anti-aging effect of SID-PEMFPeps is mediated through a multi-faceted, system-wide reprogramming of cellular functions, underscoring the power of multi-omics technologies in elucidating complex bioactivities. While these findings position SID-PEMFPeps as a promising multi-target candidate for functional foods, it is explicitly noted that translation to humans requires in vivo confirmation and further studies on safety and bioavailability.

## Figures and Tables

**Table 1:** Analysis of variance (ANOVA) of PEMFP.

Source	Sum of Squares	df	Mean Square	*F* Value	*p* Value Prob > F	
Model	815.96	14	58.28	68.11	<0.0001	***
A	79.76	1	79.76	93.20	<0.0001	***
B	30.47	1	30.47	35.61	<0.0001	***
C	44.53	1	44.53	52.04	<0.0001	***
D	2.51	1	2.51	2.93	0.1090	
AB	37.04	1	37.04	43.28	<0.0001	***
AC	7.31	1	7.31	8.54	0.0111	*
AD	51.36	1	51.36	60.02	<0.0001	***
BC	4.94	1	4.94	5.77	0.0308	*
BD	34.80	1	34.80	40.66	<0.0001	***
CD	0.14	1	0.14	0.17	0.6875	
A^2^	439.02	1	439.02	513.03	<0.0001	***
B^2^	66.29	1	66.29	77.46	<0.0001	***
C^2^	56.79	1	56.79	66.36	<0.0001	***
D^2^	150.72	1	150.72	176.13	<0.0001	***
Residual	11.98	14	0.86			
Lack of Fit	10.77	10	1.08	3.55	0.1167	not significant
Pure Error	1.21	4	0.30			
Cor Total	827.94	28				

*** represented a highly significant difference, *p* < 0.0001. * represented a statistically significant difference, *p* < 0.05.

**Table 2:** Analysis of variance (ANOVA) of PEMFPeps.

Source	Sum of Squares	df	Mean Square	F Value	*p* Value Prob > F	
Model	119.72	14	8.55	473.43	<0.0001	***
A	1.38	1	1.38	76.42	<0.0001	***
B	1.24	1	1.24	68.60	<0.0001	***
C	5.63 × 10^−5^	1	5.63 × 10^−5^	3.119 × 10^−3^	0.9563	
D	3.30	1	3.30	182.88	<0.0001	***
AB	0.69	1	0.69	38.09	<0.0001	***
AC	0.19	1	0.19	10.40	0.0061	**
AD	2.46	1	2.46	136.46	<0.0001	***
BC	0.56	1	0.56	30.77	<0.0001	***
BD	0.63	1	0.63	34.99	<0.0001	***
CD	4.66	1	4.66	258.06	<0.0001	***
A2	13.35	1	13.35	739.20	<0.0001	***
B2	64.22	1	64.22	3555.28	<0.0001	***
C2	35.48	1	35.48	1964.19	<0.0001	***
D2	41.81	1	41.81	2314.49	<0.0001	***
Residual	0.25	14	0.018			
Lack of Fit	0.18	10	0.018	1.01	0.5431	not significant
Pure Error	0.072	4	0.018			
Cor Total	119.97	28				

*** represented a highly significant difference, *p* < 0.0001. ** represented a statistically significant difference, *p* < 0.01.

## Data Availability

All data supporting the findings of this study are available in the article and its Supplementary Materials. Further inquiries can be directed to the corresponding author.

## Supplementary Materials

The following supporting information is available online at: https://www.mdpi.com/article/10.3390/foods14223977/s1, including: Figure S1. Flowchart of Theoretical and Experimental Approaches; Figure S2. Molecular Weight Distribution of PEMFPeps by HPLC; Figure S3. LC-MS/MS Profile of PEMFPeps (A) and SID-PEMFPeps (B); Table S1. Main Peptide Sequences of PEMFPeps; Table S2. Main Peptide Sequences of SID-PEMFPeps; Table S3. Overview of RNA-Seq statistics; Table S4. Summary of main KEGG pathways enriched in the transcriptomic analysis; Table S5. KEGG Main Pathway Statistics in Metabolomics.

## References

[1] Wu X., Liu S., Hu L., Ye Q., Wang J. (2023). Research progress on the biological activities of *Pleurotus eryngii* polysaccharide. Edible Med. Mushrooms.

[2] Lee D.E., Shin G.R., Lee S., Jang E.S., Shin H.W., Moon B.S., Lee C.H. (2016). Metabolomics reveal that amino acids are the main contributors to antioxidant activity in wheat and rice gochujangs (Korean fermented red pepper paste). Food Res. Int..

[3] Akyüz M., Kirbag S. (2009). Nutritive Value of *Pleurotus eryngii* (DC. ex Fr.) Quel. var. eryngii Grown on Various Agro-Wastes. Philipp. Agric..

[4] Biao Y., Chen X., Wang S., Chen G., Mcclements D.J., Zhao L. (2020). Impact of mushroom (*Pleurotus eryngii*) flour upon quality attributes of wheat dough and functional cookies-baked products. Food Sci. Nutr..

[5] Yuan B., Zhao L., Rakariyatham K., Han Y., Gao Z., Kimatu B.M., Hu Q., Xiao H. (2017). Isolation of a novel bioactive protein from an edible mushroom *Pleurotus eryngii* and its anti-inflammatory potential. Food Funct..

[6] Nie Y., Zhang P., Deng C., Xu L., Yu M., Yang W., Zhao R., Li B. (2019). Effects of *Pleurotus eryngii* (mushroom) powder and soluble polysaccharide addition on the rheological and microstructural properties of dough. Food Sci. Nutr..

[7] Li Y.B., Yang Y.L., Chen J.C., Shen H.S., Lai P.F. (2014). Nutritional Analysis and Evaluation for Industrial Cultivated Pleurotus eryngii By-products. Fujian J. Agric. Sci..

[8] Ghorai S., Banik S.P., Verma D., Chowdhury S., Mukherjee S., Khowala S. (2009). Fungal biotechnology in food and feed processing. Food Res. Int..

[9] Han Q., Li H., Zhao F., Gao J., Liu X., Ma B. (2023). *Auricularia auricula* Peptides Nutritional Supplementation Delays H(2)O(2)-Induced Senescence of HepG2 Cells by Modulation of MAPK/NF-κB Signaling Pathways. Nutrients.

[10] Aursuwanna T., Noitang S., Sangtanoo P., Srimongkol P., Saisavoey T., Puthong S., Reamtong O., Karnchanatat A. (2022). Investigating the cellular antioxidant and anti-inflammatory effects of the novel peptides in lingzhi mushrooms. Heliyon.

[11] Geng X., Tian G., Zhang W., Zhao Y., Zhao L., Wang H., Ng T.B. (2016). A Tricholoma matsutake Peptide with Angiotensin Converting Enzyme Inhibitory and Antioxidative Activities and Antihypertensive Effects in Spontaneously Hypertensive Rats. Sci. Rep..

[12] Li M., Lv R., Wang C., Ge Q., Du H., Lin S. (2021). Tricholoma matsutake-derived peptide WFNNAGP protects against DSS-induced colitis by ameliorating oxidative stress and intestinal barrier dysfunction. Food Funct..

[13] Li H., Gao J.a., Zhao F., Liu X., Ma B. (2023). Bioactive Peptides from Edible Mushrooms—The Preparation, Mechanisms, Structure—Activity Relationships and Prospects. Foods.

[14] Zhao F., Gao J., Li H., Huang S., Wang S., Liu X. (2024). Identification of Peptides from Edible *Pleurotus eryngii* Mushroom Feet and the Effect of Delaying D-Galactose-Induced Senescence of PC12 Cells Through TLR4/NF-κB/MAPK Signaling Pathways. Foods.

[15] Xinlei Z. (2019). Study on Preparation, Physicochemicai Properties and Antioxidant Activity of Pleurotus eryngi Isolated Protein. Master’s Theses.

[16] Qiancheng L.Q.L.C.G.Z.Z. (2013). Optimization of Preparation of Protein fro m *Pleurotus eryngii* via Alkali Extraction and Acid Precipitation. Food Ind..

[17] Bonan N. (2014). Research of Prepration and Antioxidant activity of Xinjiang Pleurotus FerulaeLenzi Peptides. Master’s Theses.

[18] Ning X. (2012). Preparation and Function of Protein from Rice Bran and Broken Rice Using Hydrothermal Cooking. Ph.D. Thesis.

[19] Wenkai H. (2007). Study on Preparation of Immuno-Activated Soybean Peptide Using Membrane Seperation. Master’s Theses.

[20] Jiefa Z.H.W.H.C.Y.Z. (2021). Research on the Extraction Process of Proteins from the Stipes of *Pleurotus eryngii*. Sci. Consult. (Sci. Technol. Manag.).

[21] Zhou H.C., Li H., Zhao D., Zhang W.M., Zhang J., Liu X.Y., Liu X.Q. (2020). Optimization of Protein Extraction Process of Flaxseed Cake and Study on Its Hydrolyzate Inhibitory Activity of α-Amylase. Food Res. Dev..

[22] Taofiq O., Silva A.R., Costa C., Ferreira I., Nunes J., Prieto M.A., Simal-Gandara J., Barros L., Ferreira I.C.F.R. (2020). Optimization of ergosterol extraction from *Pleurotus mushrooms* using response surface methodology. Food Funct..

[23] Huanwei Z. (2019). Study on Preparation Technology and the Activity of Polypeptide from Pleurotus eryngii In Vitro. Master’s Theses.

[24] (2023). Determination of Crude Polysaccharide Content in Edible Mushrooms - Spectrophotometric Method.

[25] (2009). Determination of Total Sugar Content in Edible Mushrooms.

[26] (2016). National Food Safety Standard—Determination of Fat in Food.

[27] (2016). National Food Safety Standard—Determination of Ash in Food.

[28] (2016). National Food Safety Standard—Determination of Protein in Food.

[29] Amakye W.K., Hou C., Xie L., Lin X., Gou N., Yuan E., Ren J. (2021). Bioactive anti-aging agents and the identification of new anti-oxidant soybean peptides. Food Biosci..

[30] (2016). National Food Safety Standard: Determination of Amino Nitrogen in Foods.

[31] Brodkorb A., Egger L., Alminger M., Alvito P., Assunção R., Ballance S., Bohn T., Bourlieu-Lacanal C., Boutrou R., Carrière F. (2019). INFOGEST static in vitro simulation of gastrointestinal food digestion. Nat. Protoc..

[32] Chauhan B., Gupta R. (2004). Application of statistical experimental design for optimization of alkaline protease production from *Bacillus* sp. RGR-14. Process. Biochem..

[33] Campisi J. (2013). Aging, cellular senescence, and cancer. Annu. Rev. Physiol..

[34] Rahman I., MacNee W. (2000). Oxidative stress and regulation of glutathione in lung inflammation. Eur. Respir. J..

[35] Mattson M.P. (2004). Pathways towards and away from Alzheimer’s disease. Nature.

[36] Mauvais-Jarvis F. (2011). Estrogen and androgen receptors: Regulators of fuel homeostasis and emerging targets for diabetes and obesity. Trends Endocrinol. Metab..

[37] Steffen K.K., Dillin A. (2016). A Ribosomal Perspective on Proteostasis and Aging. Cell Metab..

[38] Martinovic J., Zaric Kontic M., Dragic M., Todorovic A., Gusevac Stojanovic I., Mitrovic N., Grkovic I., Drakulic D. (2023). Chronic oral d-galactose intake provokes age-related changes in the rat prefrontal cortex. Behav. Brain Res..

[39] Cao P., Zhang J., Huang Y., Fang Y., Lyu J., Shen Y. (2019). The age-related changes and differences in energy metabolism and glutamate-glutamine recycling in the d-gal-induced and naturally occurring senescent astrocytes in vitro. Exp. Gerontol..

[40] Nam S.M., Seo M., Seo J.S., Rhim H., Nahm S.S., Cho I.H., Chang B.J., Kim H.J., Choi S.H., Nah S.Y. (2019). Ascorbic Acid Mitigates D-galactose-Induced Brain Aging by Increasing Hippocampal Neurogenesis and Improving Memory Function. Nutrients.

[41] Yue T., Tan H., Shi Y., Xu M., Luo S., Weng J., Xu S. (2022). Serum Metabolomic Profiling in Aging Mice Using Liquid Chromatography—Mass Spectrometry. Biomolecules.

[42] Vamecq J., Andreoletti P., El Kebbaj R., Saih F.E., Latruffe N., El Kebbaj M.H.S., Lizard G., Nasser B., Cherkaoui-Malki M. (2018). Peroxisomal Acyl-CoA Oxidase Type 1: Anti-Inflammatory and Anti-Aging Properties with a Special Emphasis on Studies with LPS and Argan Oil as a Model Transposable to Aging. Oxidative Med. Cell. Longev..

[43] Guest P. (2019). Reviews on Biomarker Studies in Aging and Anti-Aging Research.

[44] Noulala C.G.T., Ouete J.L.N., Atangana A.F., Mbahbou G.T.B., Fotso G.W., Stammler H.-G., Lenta B.N., Happi E.N., Sewald N., Ngadjui B.T. (2022). Soyauxinine, a New Indolopyridoquinazoline Alkaloid from the Stem Bark of *Araliopsis soyauxii* Engl. (Rutaceae). Molecules.

[45] Chen H., Zhou X., Hu J., Li S., Wang Z., Zhu T., Cheng H., Zhang G. (2023). Genetic insights into the association of statin and newer nonstatin drug target genes with human longevity: A Mendelian randomization analysis. Lipids Health Dis..

[46] Plainfossé H., Burger P., Herbette G., Bertrand S., Verger-Dubois G., Azoulay S., Landreau A., Papaiconomou N., Fernandez X. (2023). Anti-inflammatory and anti-aging potential of extracts and constituents from *Teucrium lucidum* L. aerial parts. J. Pharmacogn. Phytochem..

[47] Patel M.S., Korotchkina L.G. (2003). The biochemistry of the pyruvate dehydrogenase complex. Biochem. Mol. Biol. Educ..

[48] Stincone A., Prigione A., Cramer T., Wamelink M.M., Campbell K., Cheung E., Olin-Sandoval V., Grüning N.M., Krüger A., Tauqeer Alam M. (2015). The return of metabolism: Biochemistry and physiology of the pentose phosphate pathway. Biol Biol. Rev..

[49] He M., Zhou X., Wang X. (2024). Glycosylation: Mechanisms, biological functions and clinical implications. Signal Transduct. Target. Ther..

[50] Reily C., Stewart T.J., Renfrow M.B., Novak J. (2019). Glycosylation in health and disease. Nat. Rev. Nephrol..

[51] Krugmann S., Andrews S., Stephens L., Hawkins P.T. (2006). ARAP3 is essential for formation of lamellipodia after growth factor stimulation. J. Cell Sci..

[52] Ladagu A.D., Olopade F.E., Adejare A., Olopade J.O. (2023). GluN2A and GluN2B N-Methyl-D-Aspartate Receptor (NMDARs) Subunits: Their Roles and Therapeutic Antagonists in Neurological Diseases. Pharmaceuticals.

[53] Chen X.Y., Du Y.F., Chen L. (2018). Neuropeptides Exert Neuroprotective Effects in Alzheimer’s Disease. Front. Mol. Neurosci..

[54] Logan C.Y., Nusse R. (2004). The Wnt signaling pathway in development and disease. Annu. Rev. Cell Dev. Biol..

[55] Li X., Wang P., Liu M., Xu X., Wang G., Zhong B. (2025). Research Progress on Bioactivity and Extraction Technology of Different Components from Hericium erinaceus. Agric. Prod. Process..

[56] Zhu M., Feng Y., Li M., Yang Y. (2025). Optimization of Extraction Process of Protein from *Coprinus comatus* and Study on Its Antiox-idant Activity. China Condiment.

[57] Pownall T.L., Udenigwe C.C., Aluko R.E. (2010). Amino acid composition and antioxidant properties of pea seed (*Pisum sativum* L.) enzymatic protein hydrolysate fractions. J. Agric. Food Chem..

