# Supplementary material for: Production and Optimization of Anti-Aging Peptides from Pleurotus eryngii Mushroom Feet: Mechanistic Insights via Integrated Transcriptomics and Metabolomics
Source: Foods. 2025 Nov 20;14(22):3977. doi: 10.3390/foods14223977 (PMC12652877; doi:10.3390/foods14223977)
Supplement: Supplementary file 1 [file foods-14-03977-s001.zip › Supplementary Figure.pdf]

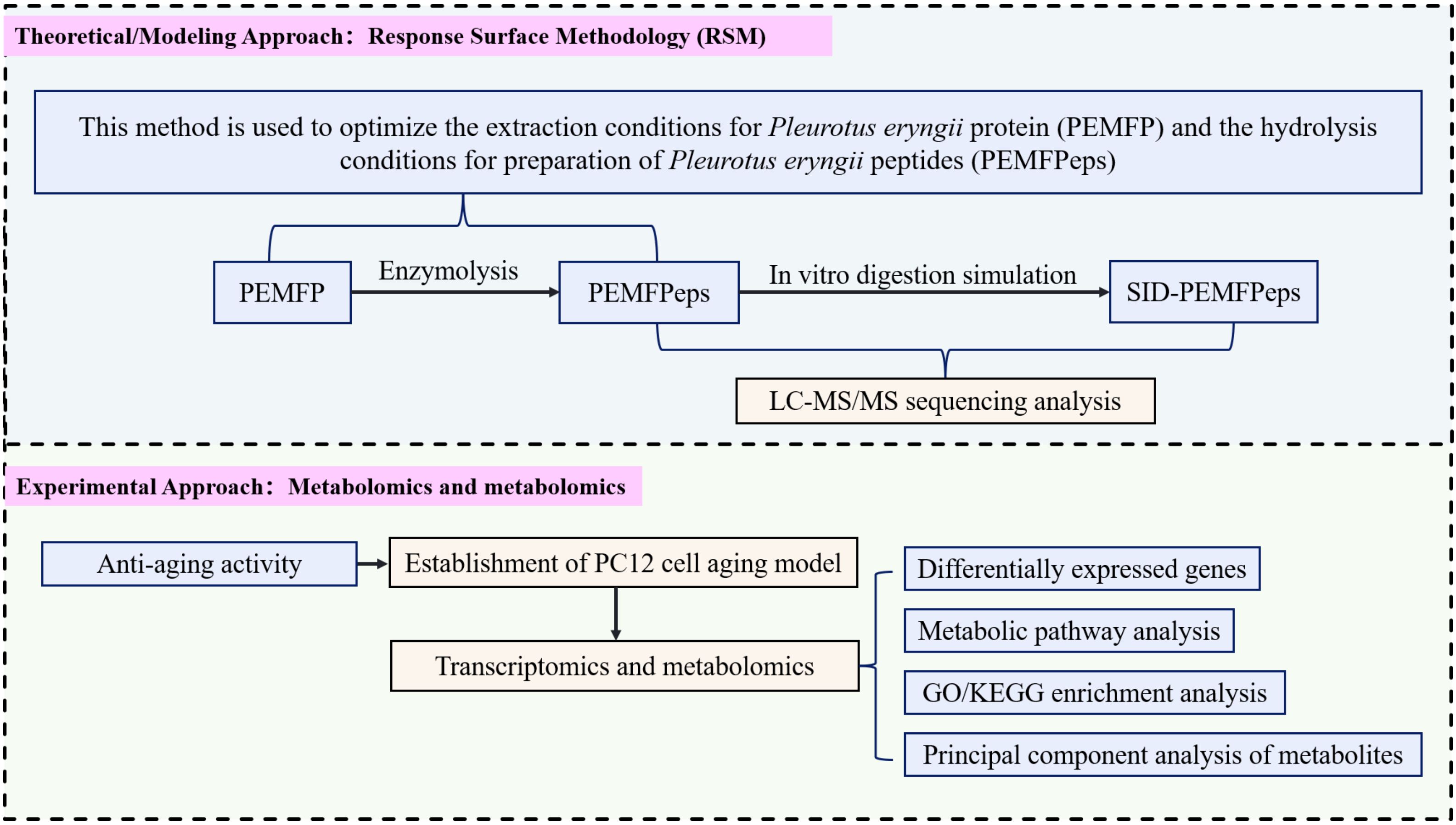

Figure S1. Flowchart of Theoretical and Experimental Approaches

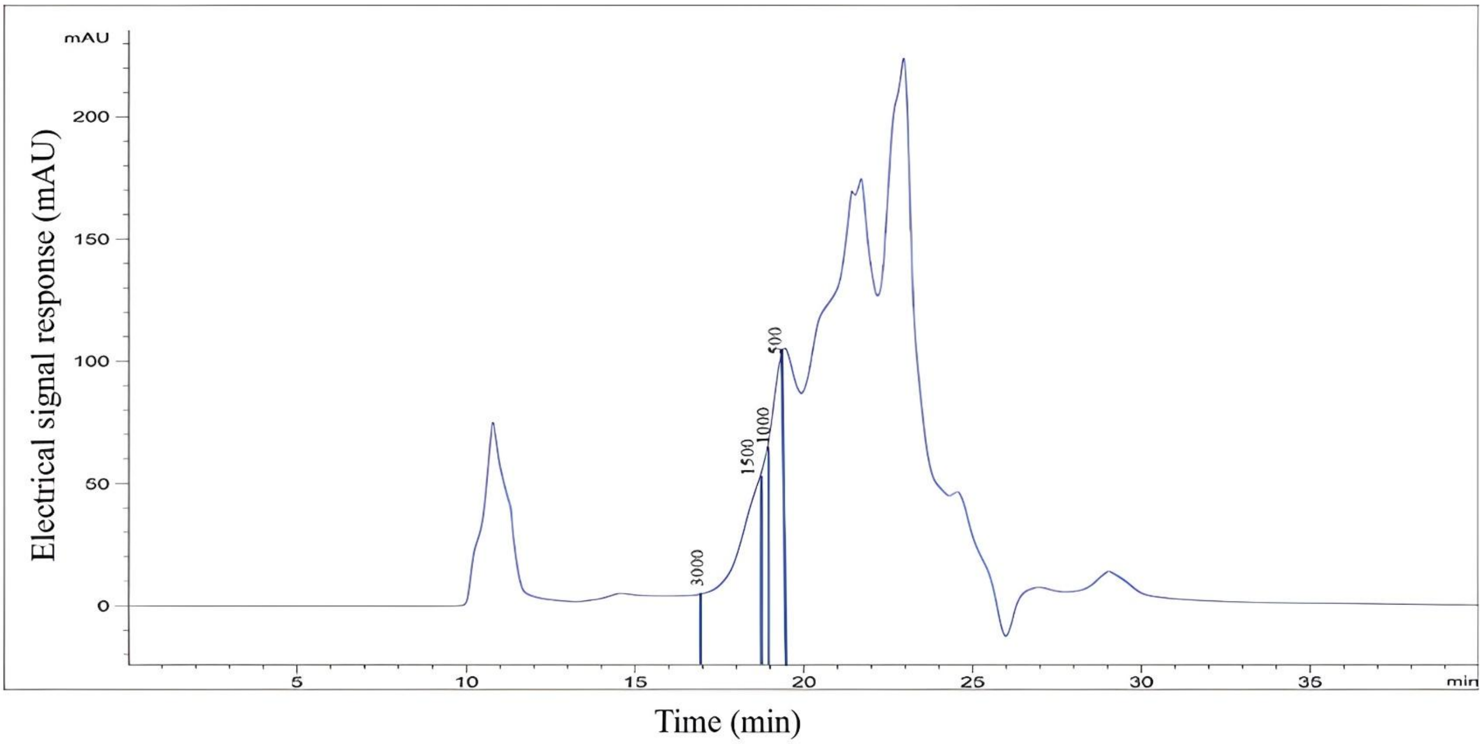

Figure S2. Molecular Weight Distribution of PEMFPeps by HPLC. Standards are insulin (5733 Da), bacitracin (1422 Da), Gly-Gly-Tyr-Arg (451 Da), and Gly-Gly-Gly (189 Da), respectively. In PEMFPeps, approximately 9.71% of the molecules have a molecular weight greater than 3000 Da; 1.14% are between 1500 and 3000 Da; 2.3% are between 500 and 1000 Da; and approximately 86% have a molecular weight less than 500 Da. Around 89.15% of the molecules are below 1000 Da.

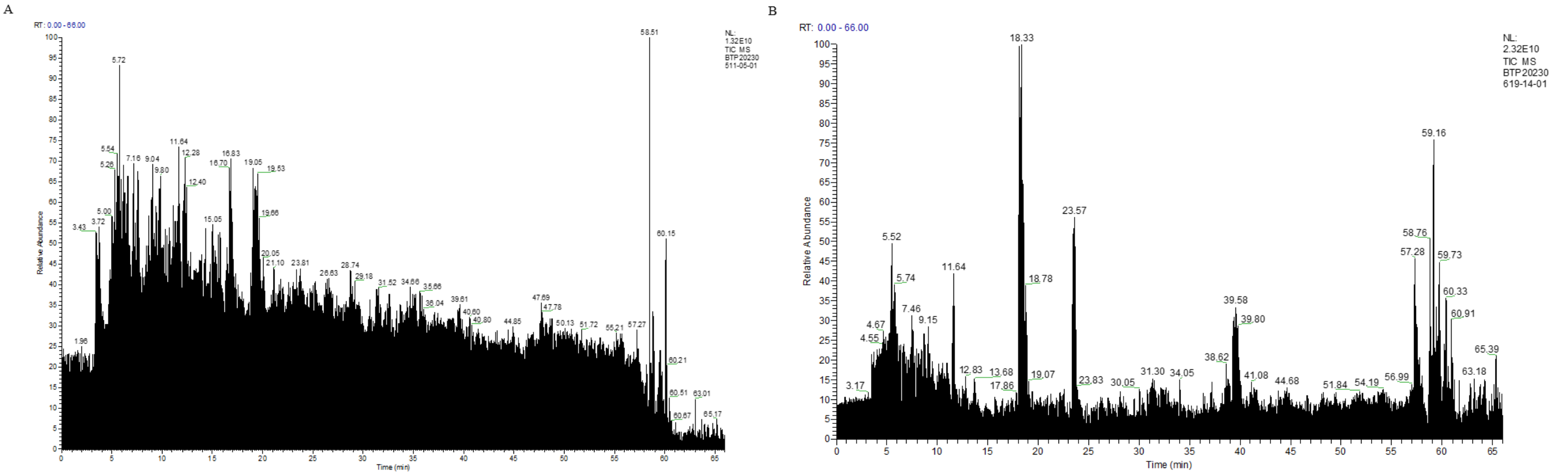

Figure S3. LC-MS/MS Profile of PEMFPeps (A) and SID-PEMFPeps (B)
