# Supplementary material for: Production and Optimization of Anti-Aging Peptides from Pleurotus eryngii Mushroom Feet: Mechanistic Insights via Integrated Transcriptomics and Metabolomics
Source: Foods. 2025 Nov 20;14(22):3977. doi: 10.3390/foods14223977 (PMC12652877; doi:10.3390/foods14223977)
Supplement: Supplementary file 1 [file foods-14-03977-s001.zip › Supplementary Table.pdf]

Table S1. Main Peptide Sequences of PEMFPeps

Peptide Sequence	Molecular Weight (Da)	Intensity	Amino Acid Number	Hydrophobic Amino Acid Number	Aromatic Amino Acid Number	Polar Amino Acid Number	Total Number of Key Amino Acids	Score
GHGFEGVTH	940.425	1.37×10^{7}	9	5	1	2	7	636.4
GRHTGPGKR	965.537	1.57×10^{8}	9	4	0	4	8	601.4
DDESAIGIR	975.474	1.64×10^{7}	9	4	0	1	5	569.6
HTGNIPLDE	995.477	1.14×10^{7}	9	4	0	1	5	564.9
DACLPSPK	887.427	8.88×10^{7}	8	4	0	1	5	564.2
GPPGTGKTL	827.460	2.90×10^{7}	9	6	0	1	7	557.3
SDEQISLK	919.474	7.45×10^{6}	8	2	0	1	3	555.8

Table S2. Main Peptide Sequences of SID-PEMFPeps

Peptide Sequence	Molecular Weight (Da)	Intensity	Amino Acid Number	Hydrophobic Amino Acid Number	Aromatic Amino Acid Number	Polar Amino Acid Number	Total Number of Key Amino Acids	Score
LLLH	495.328	3.27×10^{7}	4	3	0	1	4	336.9
VPIIPL	651.442	2.60×10^{7}	6	6	0	0	6	319.0
HVPL	465.282	1.29×10^{8}	4	3	0	1	4	314.6
PIIPL	552.375	3.73×10^{7}	5	5	0	0	5	266.1
LLLHI	608.413	6.95×10^{6}	5	4	0	1	5	255.2
LHPL	479.297	2.91×10^{7}	4	3	0	1	4	251.5
ELGVPI	627.370	2.26×10^{7}	6	5	0	0	5	242.5

Table S3. Overview of RNA-Seq statistics

Sample	Raw reads	Raw bases	Clean reads	Clean bases	Error rate (%)	Q20 (%)	Q30 (%)	GC content (%)
The control1	54,738,360	8,265,492,360	54,381,714	8,162,191,277	0.0245	98.22	94.72	49.75
The control2	42,873,646	6,473,920,546	42,586,632	6,398,138,024	0.0244	98.24	94.74	48.91
The control3	54,045,684	8,160,898,284	53,733,766	8,069,947,349	0.0241	98.39	95.13	49.64
The model1	44,859,876	6,773,841,276	44,615,698	6,705,956,500	0.0242	98.33	94.94	48.3
The model2	46,626,206	7,040,557,106	46,304,362	6,951,070,526	0.0247	98.15	94.5	49.34
The model3	53,829,866	8,128,309,766	53,458,046	8,023,311,035	0.0244	98.23	94.74	49.84
The SID-PEMFPeps-H1	50,069,346	7,560,471,246	49,735,456	7,478,942,683	0.0248	98.08	94.33	48.96
The SID-PEMFPeps-H2	54,015,608	8,156,356,808	53,666,842	8,063,965,154	0.0244	98.24	94.78	49.43
The SID-PEMFPeps-H3	47,371,588	7,153,109,788	47,025,248	7,058,743,378	0.0243	98.28	94.85	49.81

Raw reads: total number of entries of raw sequencing data; Raw bases: total data volume of raw sequencing; Clean reads: total number of entries of post-quality the control sequencing data; Clean bases: total data volume of post-quality the control sequencing; Error rate (%): average error rate of sequencing bases corresponding to the quality the control data; Q20 (%): the percentage of bases with sequencing quality above 99% of the total bases; Q30 (%): the percentage of bases with sequencing quality above 99.9% of the total bases; GC content (%): the percentage of the sum of G and C bases corresponding to QC data to the total bases.

Table S4. Summary of main KEGG pathways enriched in the transcriptomic analysis

Pathway	Pathway ID	First category	Second category	Number
Steroid biosynthesis	rno00100	Metabolism	Lipid metabolism	6
Steroid hormone biosynthesis	rno00140	Metabolism	Lipid metabolism	4
Retinol metabolism	rno00830	Metabolism	Metabolism of cofactors and vitamins	4
Drug metabolism - cytochrome P450	rno00982	Metabolism	Xenobiotics biodegradation and metabolism	3
Regulation of actin cytoskeleton	rno04810	Cellular Processes	Cell motility	2
Herpes simplex virus 1 infection	rno05168	Human Diseases	Infectious disease: viral	2
MAPK signaling pathway	rno04010	Environmental Information Processing	Signal transduction	2
B cell receptor signaling pathway	rno04662	Organismal Systems	Immune system	2
Amyotrophic lateral sclerosis	rno05014	Human Diseases	Neurodegenerative disease	2
Purine metabolism	rno00230	Metabolism	Nucleotide metabolism	1
cGMP-PKG signaling pathway	rno04022	Environmental Information Processing	Signal transduction	1
Apelin signaling pathway	rno04371	Environmental Information Processing	Signal transduction	1
Lipid and atherosclerosis	rno05417	Human Diseases	Cardiovascular disease	1
Non-alcoholic fatty liver disease	rno04932	Human Diseases	Endocrine and metabolic disease	1
Gastric cancer	rno05226	Human Diseases	Cancer: specific types	1
Staphylococcus aureus infection	rno05150	Human Diseases	Infectious disease: bacterial	1
Axon guidance	rno04360	Organismal Systems	Development and regeneration	1
IL-17 signaling pathway	rno04657	Organismal Systems	Immune system	1
Bile secretion	rno04976	Organismal Systems	Digestive system	1

Pathway ID: pathway number; Pathway: specific description of KEGG pathway; First category: 7 branches of KEGG metabolic pathway; Second category: name of KEGG metabolic pathway; Number: number of genes/transcripts enriched to the pathway.

Table S5. KEGG Main Pathway Statistics in Metabolomics

Pathway	Pathway ID	First category	Second category	Number
Glycerophospholipid metabolism	map00564	Metabolism	Lipid metabolism	115
Retrograde endocannabinoid signaling	map04723	Organismal Systems	Nervous system	72
Choline metabolism in cancer	map05231	Human Diseases	Cancer: overview	58
Kaposi sarcoma-associated herpesvirus infection	map05167	Human Diseases	Infectious disease: viral	52
Glycosylphosphatidylinositol (GPI)-anchor biosynthesis	map00563	Metabolism	Glycan biosynthesis and metabolism	43
Autophagy - animal	map04140	Cellular Processes	Transport and catabolism	43
Autophagy - other	map04136	Cellular Processes	Transport and catabolism	42
ABC transporters	map02010	Environmental Information Processing	Membrane transport	40
Purine metabolism	map00230	Metabolism	Nucleotide metabolism	26
D-Amino acid metabolism	map00470	Metabolism	Metabolism of other amino acids	23
Linoleic acid metabolism	map00591	Metabolism	Lipid metabolism	22
Protein digestion and absorption	map04974	Organismal Systems	Digestive system	21
cAMP signaling pathway	map04024	Environmental Information Processing	Signal transduction	21
Tryptophan metabolism	map00380	Metabolism	Amino acid metabolism	20
Neuroactive ligand-receptor interaction	map04080	Environmental Information Processing	Signaling molecules and interaction	20
Arachidonic acid metabolism	map00590	Metabolism	Lipid metabolism	20
alpha-Linolenic acid metabolism	map00592	Metabolism	Lipid metabolism	20
Parkinson disease	map05012	Human Diseases	Neurodegenerative disease	19
Phospholipase D signaling pathway	map04072	Environmental Information Processing	Signal transduction	19
Pathways in cancer	map05200	Human Diseases	Cancer: overview	18

Pathway ID: pathway number; Pathway: specific description of KEGG pathway; First category: 7 branches of KEGG metabolic pathway; Second category: name of KEGG metabolic pathway; Number: number of genes/transcripts enriched to the pathway.
